# A hypothalamus-habenula circuit controls aversion

**DOI:** 10.1038/s41380-019-0369-5

**Published:** 2019-02-12

**Authors:** Iakovos Lazaridis, Ourania Tzortzi, Moritz Weglage, Antje Märtin, Yang Xuan, Marc Parent, Yvonne Johansson, Janos Fuzik, Daniel Fürth, Lief E. Fenno, Charu Ramakrishnan, Gilad Silberberg, Karl Deisseroth, Marie Carlén, Konstantinos Meletis

**Affiliations:** 1 Department of Neuroscience, Karolinska Institutet, Stockholm, Sweden; 2 Department of Bioengineering, Stanford University, Stanford, CA USA

**Keywords:** Neuroscience, Physiology

## Abstract

Encoding and predicting aversive events are critical functions of circuits that support survival and emotional well-being. Maladaptive circuit changes in emotional valence processing can underlie the pathophysiology of affective disorders. The lateral habenula (LHb) has been linked to aversion and mood regulation through modulation of the dopamine and serotonin systems. We have defined the identity and function of glutamatergic (Vglut2) control of the LHb, comparing the role of inputs originating in the globus pallidus internal segment (GPi), and lateral hypothalamic area (LHA), respectively. We found that LHb-projecting LHA neurons, and not the proposed GABA/glutamate co-releasing GPi neurons, are responsible for encoding negative value. Monosynaptic rabies tracing of the presynaptic organization revealed a predominantly limbic input onto LHA Vglut2 neurons, while sensorimotor inputs were more prominent onto GABA/glutamate co-releasing GPi neurons. We further recorded the activity of LHA Vglut2 neurons, by imaging calcium dynamics in response to appetitive versus aversive events in conditioning paradigms. LHA Vglut2 neurons formed activity clusters representing distinct reward or aversion signals, including a population that responded to mild foot shocks and predicted aversive events. We found that the LHb-projecting LHA Vglut2 neurons encode negative valence and rapidly develop a prediction signal for negative events. These findings establish the glutamatergic LHA-LHb circuit as a critical node in value processing.

## Introduction

The neural circuits responsible for encoding predictions of upcoming rewards and negative events are central to motivated behaviors and the survival of animals. Limbic and basal ganglia circuits have been extensively linked to motivation, action-selection, and aversion [1]. In this context, LHb, an evolutionarily conserved epithalamic nucleus, is especially interesting due to its control over the neuromodulators dopamine and serotonin [2]. LHb neurons are activated by aversive events and inhibited by unexpected rewards, and the LHb circuitry has therefore been proposed to play an important role in signaling reward and punishment prediction errors [3, 4]. Furthermore, maladaptive changes in the LHb circuitry have been linked to depression and addiction [5–8]. In summary, the LHb has been implicated in controlling emotional status, response to stress and helplessness, and in accordance, represents a key brain structure central to understanding circuit dysfunction in affective disorders. Defining the mechanisms underlying LHb activity, and more specifically the role of the different inputs that modulate LHb activity, are important aspects in deciphering how aversive and rewarding events are represented.

The GPi, also known as the entopeduncular nucleus in rodents, contains LHb-projecting neurons [9, 10]. The GPi has classically been defined as a GABAergic output nucleus [11], but recent studies have demonstrated that the GPi also contains glutamatergic neurons [12, 13]. The primate GPi contains both reward positive and reward negative neurons [10], including glucose-sensitive neurons [14]. GPi neurons projecting to LHb can encode negative and aversive stimuli [10, 13] and co-release GABA and glutamate [12, 13, 15]. Supporting the role of the GPi-LHb pathway in aversion, optogenetic stimulation of GPi projections to LHb has been shown to control valence and to generate strong aversive responses [13, 16].

The hypothalamus has been extensively studied in the regulation of basic behaviors, such as feeding, sexual reproduction and aggression [17–19]. The lateral region of hypothalamus (LHA) is a functionally heterogeneous structure that influences cognitive, emotional, motor, and autonomic functions [20–22]. Electrical self-stimulation experiments have demonstrated the importance of the LHA in positive reinforcement [23, 24]. The role of the LHA in reward processing is supported by findings of inputs to the LHA from a number of corticolimbic structures [25]. The LHA itself sends a glutamatergic projection to the LHb [26, 27]. The diverse input system, and functionality of the LHA [28], indicate complex cell type-specific LHA signals in the shaping of motivated behaviors. Overall, the relationship between the LHA circuitry, including a potentially large number of unidentified neuron subtypes, and animal behavior has not been resolved [22, 29].

To delineate the source of negative valence signals to LHb we have in the current study characterized the LHb-projecting glutamatergic (Vglut2) population located in the GPi/LHA border region. We have compared the properties, circuit organization, and function, of two main populations; the LHb-projecting GABA/glutamate co-releasing population located in the GPi, and the LHb-projecting glutamatergic population in the LHA. In order to genetically target the two neuron types, we used RNA sequencing from single neuron nuclei to identify differential markers for the GABA/glutamate co-releasing GPi neurons, and other Vglut2 neurons in the GPi/LHA border region. The neuronal populations were thereafter genetically targeted in mouse lines, and their role in shaping aversive behavior and the value of actions was defined. We found that GABA/glutamate co-releasing GPi-LHb neurons do not generate aversive behaviors, but in contrast, activation of LHA Vglut2 neurons produces strong aversive responses. Furthermore, LHA Vglut2 neurons, again in contrast to the GABA/glutamate co-releasing GPi-LHb neurons, also shape the value of actions. Based on these results, we performed in vivo imaging to identify candidate LHA Vglut2 populations that carry prediction signals for punishment or aversion, reflecting the neural signals expected to be found in aversion-related excitatory inputs to LHb. Imaging the in vivo activity of Vglut2 neurons in LHA revealed several functional clusters with distinct responses to rewards and aversion, including neurons that displayed a response profile that is consistent with a role in encoding negative valence and driving aversive behavior through LHb. Through a genetic intersectional approach, we imaged specifically the activity of LHb-projecting LHA Vglut2 neurons, and found that this population encodes the aversive event in fear conditioning, and importantly, also rapidly develops a prediction signal for the upcoming negative event.

In summary, we have defined the role of GPi versus LHA projections to LHb in aversive behavior and action value coding, and we have found that a novel glutamatergic LHA-LHb population is responsible for signaling as well as predicting future negative events.

## Results

### Whole-brain mapping of Vglut2 neurons projecting to LHb

**Fig. 1:**
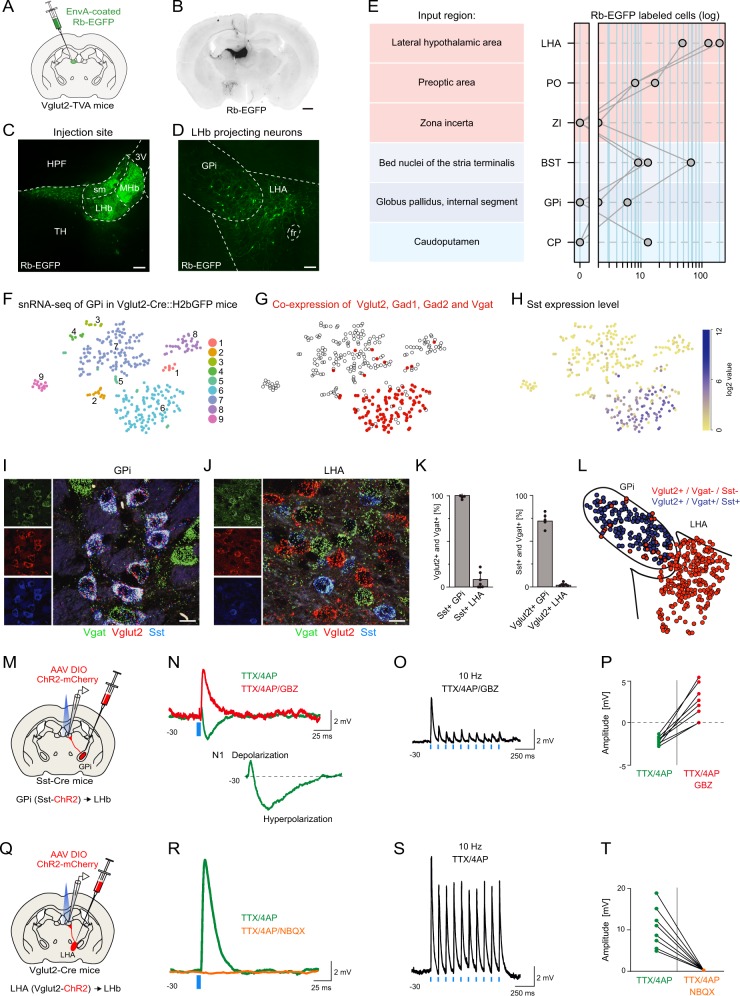
Molecular and functional separation of the GPi-LHb and LHA-LHb pathways. **a** Illustration of the experimental approach. EnvA-coated Rb-EGFP virus was injected into the LHb in Vglut2-TVA mice. **b** Coronal section showing Rb-EGFP labeled neurons (black) in the injection site (LHb) and input regions. **c** Rb-EGFP labeling in the injection site (LHb). **d** Rb-EGFP labeling of Vglut2 + LHb-projecting neurons in the GPi and LHA region. See supplementary Fig. S1 for detailed mapping of Rb-EGFP labeled LHb-projecting neurons. **e** Quantification of the distribution of Rb-EGFP-labeled input neurons in basal ganglia (blue hues) and the hypothalamus (pink hues) (*n* = 3 Vglut2-TVA mice). **f**–**h** Molecular identity of Vglut2 + neurons in the GPi as revealed by snRNA-seq in Vglut2-Cre::H2bGFP mice. **f** t-SNE visualization of the transcriptome of single Vglut2 + neuron nuclei in the GPi region (*n* = 312 nuclei). **g** As **f**. Single nuclei co-expressing the four genes Vglut2, Gad1, Gad2, and Vgat are marked (red). **h** As **f**. Sst expression levels in individual nuclei is shown. **i** Representative image of in situ hybridization showing Vgat, Vglut2, and Sst co-expression in single GPi neurons. **j** Representative image of in situ hybridization showing LHA Vglut2 neurons lack expression of Vgat and Sst. **k** Left: quantification of the co-expression of Vglut2 and Vgat in Sst + neurons in the GPi and LHA (in situ hybridization, *n* = 647 neurons, 6 sections from 3 mice). Right: quantification of the co-expression of Sst and Vgat in Vglut2 + neurons in the GPi and LHA (in situ hybridization, *n* = 1292 neurons, 7 sections from 3 mice). **l** Anatomical distribution of Vglut2 + /Vgat + /Sst + (blue) and Vglut2 + /Vgat-/Sst- (red) neurons in the GPi-LHA border region. Data from four superimposed representative coronal sections (*n* = 449 neurons). See supplementary Fig. S2 and Fig. S3 for detailed mapping of cell types and spatial definition of the GPi-LHA border. **m** Illustration of the strategy for optogenetic characterization of monosynaptic inputs from GPi Sst neurons projecting to the LHb. AAV DIO ChR2-mCherry (red) injection into the GPi of Sst-Cre mice labels projections to the LHb. Blue light (470 nm) activates ChR2-expressing terminals, while postsynaptic potentials in LHb neurons are recorded in whole-cell mode. **n** Representative monosynaptic response in a LHb neuron after optogenetic activation of Sst + GPi terminals (green trace). Bath application of the GABA-A antagonist gabazine (GBZ; red trace) abolished the hyperpolarizing inhibitory component resulting in a larger positive excitatory component. Baseline postsynaptic response shown in the insert (N1). **o** Representative example of short-term synaptic depression of the monosynaptic responses in a LHb neuron after optogenetic stimulation of Sst + GPi inputs to LHb (10 Hz, 5 ms pulse, 10 pulses; blue bars). **p** Quantification of the peak amplitude of the inhibitory (green) and excitatory (red) component of the postsynaptic response in LHb neurons (*n* = 8) before and after GBZ (*P* < 0.001). **q** Illustration of the strategy for optogenetic characterization of monosynaptic inputs from LHA Vglut2 neurons projecting to the LHb. AAV DIO ChR2-mCherry (red) injection into the LHA of Vglu2-Cre mice labels projections to the LHb. Blue light (470 nm) activates ChR2, while postsynaptic potentials in LHb neurons are recorded in whole-cell mode. **r** Representative monosynaptic response in a LHb neuron after optogenetic activation of LHA Vglut2 axon terminals (green trace). The excitatory response was abolished by NBQX (orange trace), demonstrating a direct glutamatergic synapse. No inhibitory component was detected. **s** Representative example of monosynaptic responses in a LHb neuron evoked by optogenetic stimulation of the axon terminals of LHA Vglut2 neurons (10 Hz, 5 ms pulse, 10 pulses; blue bars). **t** Significant reduction of the peak amplitude of the excitatory response in LHb neurons in the presence of NBQX (orange trace; *P* < 0.001). Green trace: baseline (*n* = 8 neurons). Scale bars: 1 mm in (**b**), 200 µm in (**c**, **d**),10 µm in **i**, **j**. HPF Hippocampal formation, sm stria medullaris, TH thalamus, 3 V third ventricle, MHb medial habenula. Preoptic area (PO) is defined here as the combined medial preoptic area and lateral preoptic area

To identify discrete glutamatergic inputs that target LHb on a whole-brain scale, we injected an EGFP-expressing EnvA-coated rabies virus (Rb-EGFP) lacking the rabies glycoprotein G (RG) gene, into the LHb of adult mice expressing the TVA receptor in all Vglut2 neurons (Vglut2-TVA mice; Fig. 1a–d). This genetically-restricted, retrograde tracing approach is based on the uptake of the EnvA-coated rabies virus in TVA-expressing axon terminals of Vglut2 neurons at the injection site (here the LHb). Coronal brain sections were imaged, and the EGFP-labeled neurons were anatomically annotated onto a mouse reference atlas (supplementary Fig. S1). We focused the analysis on regions in the basal ganglia and the hypothalamus, and we found that the LHA was the most prominent input region to LHb in terms of absolute numbers of Rb-EGFP labeled neurons (Fig. 1e). The labeled LHA neurons were localized close to the GPi border (Fig. 1d), a region that has been shown to project negative signals to LHb [10]. The observed diversity in spatial distribution of LHb-projecting neurons is in line with earlier characterizations of LHb afferents [30].

### The identity and function of Vglut2 neurons in GPi and LHA

Since we identified LHb-projecting Vglut2 neurons in the GPi as well as the bordering LHA, we aimed to determine the possible molecular diversity of these Vglut2 neurons. To identify markers that can differentiate between Vglut2 neuron subtypes, we used RNA sequencing of single neuron nuclei (snRNA-seq) [31]. We microdissected the GPi region from adult Vglut2-Cre::H2bGFP reporter mice and sorted single GFP + nuclei using FACS. We performed snRNA-seq of 312 single nuclei, detecting on average 3502 genes per neuron nucleus. We clustered the neuron nuclei based on their gene expression profile data and visualized the different clusters using t-distributed stochastic neighbor embedding (t-SNE) [32], to uncover potential markers in the data that could support a molecular distinction between GPi and LHA Vglut2 neurons. We identified nine distinct clusters (Fig. 1f), and we focused our analysis on the neuron nuclei with co-expression of glutamatergic markers (e.g. Vglut2) and GABAergic markers (e.g. Vgat, Gad1, and Gad2), as we expected these to identify the GABA/glutamate co-releasing GPi neurons [12]. We identified one cluster (cluster 6) containing 109 nuclei with high co-expression of Vglut2, Gad1, Gad2, and Vgat (Fig. 1g). To identify other markers for these putative GABA/glutamate co-releasing neurons, we analyzed the differential gene expression profile and found a number of distinct markers, including specific expression of the Somatostatin (Sst) gene (Fig. 1h), paralleling findings in a recent study [15]. We next analyzed the anatomical distribution of the Sst + /Vgat + /Vglut2 + GPi population using multiplexed in situ hybridization [33] in adult mouse brain sections (Fig. 1i, j). Consistent with the snRNA-seq data, we found that 99% ± 1.9 s.d. of the Sst + GPi neurons co-expressed the GABA/glutamate markers Vgat and Vglut2 (Fig. 1k). In contrast, 98% ± 0.9 s.d. of the Vglut2 neurons in the LHA were negative for Sst and Vgat, and were distinctly distributed across the GPi/LHA border compared to the Vglut2 + /Vgat + /Sst + GPi neurons (Fig. 1l). Based on spatial mapping of cell specific markers, we were able to redefine the GPi/LHA border and map the distribution of the GPi-LHb pathway, with a combination of tissue mapping using in situ hybridization (supplementary Fig. S2A-C), genetic labeling of Vglut2/Vgat neurons (supplementary Fig. S2D, Fig. S3A-B), and immunostaining (supplementary Fig. S2E-H). We further confirmed that Sst can serve as an identifying marker for Vglut2/Vgat GPi neurons since retrograde labeling of Sst + neurons after injection of the Cre-dependent retrograde adeno-associated virus (rAAV DIO ChR2-mCherry) into the LHb of Sst-Cre mice only resulted in labeling of Sst + neurons in the GPi (supplementary Fig. S3C-E). In summary, the co-expression of Vglut2 and Vgat, or of Vglut2 and Sst, defines molecularly the GPi-LHb pathway, and Sst can be used as a marker to differentiate between GABA/glutamate co-releasing GPi-LHb neurons (Sst^GPi-LHb^) versus LHA-LHb-projecting Vglut2 neurons (Vglut2^LHA-LHb^).

To study the function of the GPi and LHA projections to the LHb, we performed optogenetic activation of the Sst + ^GPi-LHb^ versus Vglut2^LHA-LHb^ axon terminals together with whole-cell current clamp recordings from LHb neurons. For this, we injected the Cre-dependent adeno-associated virus (AAV DIO ChR2-mCherry) into the GPi of adult Sst-Cre mice or into the LHA of adult Vglut2-Cre mice. We pharmacologically isolated the ChR2-induced monosynaptic inputs (bath application of 1 µM TTX, 100 µM 4AP)[34], and we determined the excitatory and inhibitory postsynaptic components (NBQX 10 µM, gabazine 10 µM) after optogenetic stimulation of the ChR2 + terminals in the LHb (Fig. 1m–o). We found that optogenetic activation of Sst + ^GPi-LHb^ terminals resulted in synaptic co-release of GABA and glutamate (Fig. 1n). The net postsynaptic component had a negative amplitude of 2.1 ± 0.8 mV (TTX, 4AP), and application of a GABA-A receptor antagonist gabazine (TTX, 4AP, GBZ) revealed a larger positive excitatory component, resulting in a 2.9 ± 1.1 mV response (Fig. 1p). Co-release was found in the majority of recorded LHb neurons (6/8 neurons), although some LHb neurons showed only inhibitory responses (2/8 neurons). In contrast, optogenetic activation of the Vglut2^LHA-LHb^ ChR2-expressing terminals generated a strong excitatory response in all recorded LHb neurons (8/8 neurons, Fig. 1q–t). The positive postsynaptic potential of 10.9 ± 3.2 mV was blocked by the AMPA receptor antagonist NBQX (TTX, 4AP, NBQX) and did not reveal any inhibitory potential (Fig. 1r). Together these data support a distinct functional control of LHb neurons by the Sst +^GPi-LHb,^ and the Vglut2^LHA-LHb^ projections.

### Whole-brain organization of inputs to Vglut2^GPi-LHb^ versus Vglut2^LHA-LHb^ neurons

**Fig. 2:**
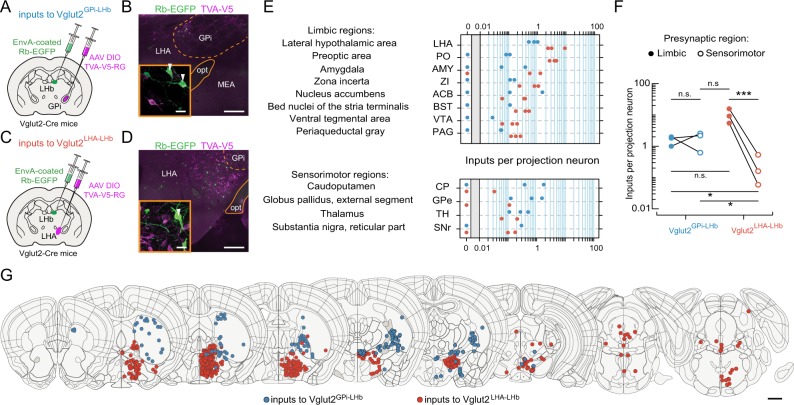
Whole-brain mapping of presynaptic inputs differentiates the GPi-LHb and LHA-LHb pathways. **a** Illustration of the experimental approach. Retrograde tracing of monosynaptic inputs to GPi Vglut2 neurons that project to LHb (Vglut2^GPi-LHb^). AAV DIO TVA-V5-RG (purple) was injected into the GPi followed by injection of EnvA-coated Rb-EGFP virus (green) into the LHb of Vglut2-Cre mice. **b** Representative image of starter neurons (white arrowhead) in the GPi, defined by co-expression of TVA-V5 (purple) and Rb-EGFP (green). **c** Illustration of experimental approach. Retrograde tracing of monosynaptic inputs to LHA Vglut2 neurons that project to LHb (Vglut2^LHA-LHb^). AAV DIO TVA-V5-RG (purple) was injected into the LHA followed by injection of EnvA-coated rabies-EGFP virus (green) into the LHb of Vglut2-Cre mice. **d** Representative image of starter neurons (white arrowhead) in the LHA, defined by co-expression of TVA-V5 (purple) and Rb-EGFP (green). **e** Quantification of the distribution of Rb-EGFP labeled presynaptic neurons according to their localization in limbic versus sensorimotor regions. Vglut2^LHA-LHb^ neurons receive significantly more inputs from limbic compared to sensorimotor regions (Vglut2^GPi-LHb^: *n* = 326 presynaptic neurons, 3 mice; Vglut2^LHA-LHb^: 2548 presynaptic neurons, 3 mice). Data points represent individual animals. **f** Comparison of the distribution of Rb-EGFP labeled presynaptic neurons found in limbic versus sensorimotor regions, for Vglut2^GPi-LHb^ neurons (blue), or Vglut2^LHA-LHb^ neurons (red). Data points represent individual animals. A two-way mixed effect ANOVA with projection pathway (Vglut2^GPi-LHb^ versus Vglut2^LHA-LHb^) as well as input region (limbic versus sensorimotor) as factors demonstrated a significant interaction effect [*F*_(1,8)_ = 21.24, *P* < 0.01]. **g** Representative whole-brain mapping of Rb-EGFP labeled presynaptic neurons (blue, presynaptic to Vglut2^GPi-LHb^ neurons, *n* = 1 mouse; red, presynaptic to Vglut2^LHA-LHb^ neurons, *n* = 1 mouse). See supplementary Fig. S4 for detailed mapping of starter neurons and Rb-EGFP labeled presynaptic neurons. Scale bars: 50 µm in **a**, **d**, 10 µm in (**a**) and (**d**) insets. *P *<* 0.05, ***P *<* 0.001

To further investigate whether the Vglut2^GPi-LHb^ and Vglut2^LHA-LHb^ populations also form distinct pathways, we mapped their respective presynaptic organization on the whole-brain level using monosynaptic retrograde rabies virus tracing [35]. To ensure co-expression of the TVA receptor (TVA fused to a V5 tag; TVA-V5) and the rabies glycoprotein (RG) in the Cre-expressing starter neurons, we generated a single AAV DIO vector expressing TVA-V5 and RG. To trace the monosynaptic inputs for Vglut2^GPi-LHb^ versus Vglut2^LHA-LHb^ neurons, we injected a small volume (70 nl) of the AAV DIO TVA-V5 in Vglut2-Cre mice targeting either GPi (Vglut2^GPi-LHb^) or the LHA (Vglut2^LHA-LHb^). A genetically modified rabies virus (EnvA-coated deltaG rabies virus, Rb-EGFP) was injected 21 days later into the LHb, thereby restricting uptake of Rb-EGFP to GPi or LHA Vglut2 neurons with TVA-expressing axon terminals in LHb (Fig. 2a–d). We determined the number and precise anatomical localization of the starter neurons based on the co-expression of TVA-V5 and EGFP (supplementary Fig. S4A). Whole-brain mapping of the presynaptic neurons for the Vglut2^GPi-LHb^ and Vglut2^LHA-LHb^ populations, revealed that the two populations received overlapping, as well as distinct inputs (Fig. 2e, supplementary Fig. S4B-C). Specifically, we found that for Vglut2^GPi-LHb^ neurons a large fraction of inputs originated in sensorimotor regions (e.g. CPu, GPe), whereas Vglut2^LHA-LHb^ neurons received a significantly larger fraction of their inputs from limbic regions (e.g. PO, AMY, BST) (Fig. 2f, g). The identification of different presynaptic inputs further prompted us to investigate the differential role of the Vglut2^GPi-LHb^ and Vglut2^LHA-LHb^ in behavior.

### The role of Vglut2^GPi-LHb^ and Vglut2^LHA-LHb^ neurons in aversive behavior

**Fig. 3:**
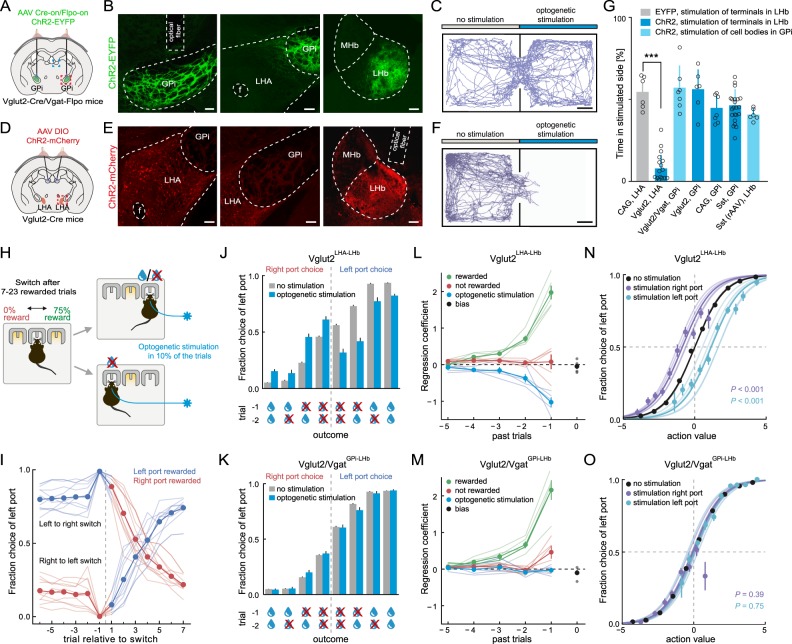
The role of Vglut2/Vgat GPi-LHb versus Vglut2 LHA-LHb neurons in aversion and action value. **a** Illustration of the experimental approach. AAV Cre-on/Flpo-on ChR2-EYFP (green) was bilaterally injected into the GPi in Vglut2-Cre/Vgat-Flpo mice to express ChR2 in GPi Vglut2/Vgat neurons. Optogenetic stimulation was targeted to ChR2 + cell bodies in the GPi. Red box: location of left and middle panels in (**b**). Blue box; location of right panel in **b**. **b** Representative image of coronal brain section, right hemisphere. Left: ChR2-EYFP expression (green) in Vglut2/Vgat neurons in the GPi. Middle: ChR2-EYFP expression (green) is limited to the GPi. Right: ChR2-EYFP expression (green) in LHb from the axon terminals of GPi Vglut2/Vgat neurons. **c** Optogenetic stimulation in the real-time place preference assay. Representative trace of the locomotion of a Vglut2-Cre/Vgat-Flpo mouse expressing ChR2-EYFP in GPi Vglut2/Vgat neurons. Blue light (447 nm) stimulation (60 Hz, 1 ms pulses) of the ChR2 + cell bodies in the GPi did not induce place aversion. **d** Illustration of the experimental approach. AAV DIO ChR2-mCherry (red) was bilaterally injected into the LHA in Vglut2-Cre mice to express ChR2 in LHA Vglut2 neurons. Optogenetic stimulation was targeted to ChR2-expressing Vglut2^LHA-LHb^ axon terminals in the LHb. Red box: location of left and middle panels in **e**. Blue box; location of right panel in **e**. **e** Representative image of coronal brain section, right hemisphere. Left: ChR2-mCherry expression (red) in LHA Vglut2 neurons. Middle: ChR2-mCherry expression (red) was targeted to the LHA, excluding the GPi. Right: ChR2-mCherry expression (red) in LHb from Vglut2^LHA-LHb^ axon terminals. **f** Optogenetic stimulation in the real-time place preference assay. Representative trace of the locomotion of a Vglut2-Cre mouse expressing ChR2-mCherry in Vglut2^LHA-LHb^ neurons. Blue light (447 nm) stimulation (60 Hz, 1 ms pulses) of the ChR2 + Vglut2^LHA-LHb^ axon terminals in the LHb induced a strong aversion to the stimulated side. **g** Quantification of place aversion in response to optogenetic stimulation of different GPi-LHb or LHA-LHb populations expressing ChR2. Fiber placement targeting axon terminals in LHb (dark blue) or cell bodies in GPi (light blue). X-axis: targeted neuronal population, injection site. CAG, LHA: *n* = 6 wildtype mice injected with AAV CAG-EYFP into LHA; Vglut2, LHA: *n* = 16 Vglut2-Cre mice injected with AAV DIO ChR2-mCherry into LHA; Vglut2/Vgat, GPi: *n* = 6 Vglut2-Cre/Vgat-Flpo mice injected with AAV Cre-on/Flpo-on-ChR2-EYFP into GPI; Vglut2, GPi: *n* = 6 Vglut2-Cre mice injected with AAV DIO ChR2-mCherry into GPi; CAG, GPi: *n* = 7 wild type mice injected with AAV CAG-ChR2 into GPi; Sst, GPi: *n* = 22 Sst-Cre mice injected with AAV DIO ChR2-mCherry into GPi; Sst (rAAV), LHb: *n* = 6 Sst-Cre mice injected with retrograde AAV DIO ChR2-mCherry into LHb. **h** Illustration of the probabilistic 2-choice switching task (for behavior details see Methods section). **i** Behavior in the probabilistic 2-choice switching task. Fraction of left port choices on trials before and after switching (gray dashed line) of which port is rewarded (*n* = 5 Vglut2-Cre mice + 5 Vglut2-Cre/Vgat-Flpo mice, same animals as in **j**–**o**. Thin lines: individual animals. Reward delivery strongly influences the choice behavior (i.e., choice of port). Blue: left port is rewarded, red: right port is rewarded. **j**, **l**, **n** Investigation of the role of Vglut2^LHA-LHb^ neurons in action value. (*n* = 5 Vglut2-Cre mice, experimental approach as in **d**. **k**, **m**, **o** Investigation of the role of Vglut2/Vgat^GPi-LHb^ neurons in action value. (*n* = 5 Vglut2-Cre/Vgat-Flpo mice, experimental approach as in **a**). **j** Choice behavior in relation to recent action value history (outcome in two trials back are shown). Past choice outcomes (action rewarded or not) strongly influence the choice behavior (gray bars). Optogenetic stimulation (447 nm, 30 Hz, 5 ms light pulses, 500 ms duration) of Vglut2^LHA-LHb^ axon terminals in the LHb (blue bars) shifts choice to the opposite port. **k** As **j**, but with optogenetic stimulation of cell bodies of Vglut2/Vgat^GPi-LHb^ neurons. Activation of Vglut2/Vgat^GPi-LHb^ neurons does not affect choice behavior. **l**, **m** Logistic regression coefficients for the effects of recent choice outcomes (green: reward was delivered, red: reward was not delivered), and the effect of optogenetic stimulation (blue) on choice behavior. Black: bias in choice. The coefficient calculation for optogenetic stimulation of Vglut2^LHA-LHb^ axon terminals in the LHb, but not for stimulation of Vglut2/Vgat^GPi-LHb^ neurons, predicts a shift in choice. Thin lines: logistic regressions fitted for individual animals. **n**, **o** Logistic regression model predicting the fraction of choices for the left port as a function of trial action value and optogenetic stimulation in the previous trial. Right and left port stimulation of Vglut2^LHA-LHb^ axon terminals in the LHb shifts the choice behavior, but not stimulation of Vglut2/Vgat^GPi-LHb^ neurons. Thick lines: single model pooling choice data from all animals. Thin lines: regressions for individual animals. Dots: actual fraction of choices for the left port binned by action value. Right and left port stimulation treated as two separate variables. Trial by trial action values calculated for each animal individually by summing the regression coefficients according to outcome history. *P* values correspond to *t*-values for the significance of the respective stimulation regression coefficient. See supplementary Fig. S3, Fig. S5, and Fig. S6 for additional information on detailed mapping of the genetic targeting of GPi neurons, cFos mapping after optogenetic stimulation, additional optogenetic data in the real-time place preference assay. See supplementary Table 1 for details on the different optogenetic strategies. Scale bars: 100 µm in **a** and **e**, 5 cm in **c** and **f**. Error bars, mean ± s.d. in **g**, mean ± s.e.m. in (H-M). ***P < 0.001

We next investigated the contribution of the Vglut2^GPi-LHb^ and Vglut2^LHA-LHb^ pathways in aversive behavior, as previous studies have identified the GPi region as a source of aversive signals [13, 16]. First, to specifically target ChR2 only to GABA/glutamate co-releasing GPi neurons, we used a double transgenic Vglut2-Cre/Vgat-Flpo mouse and injected into the GPi a Cre and Flpo -dependent ChR2 vector (AAV Cre-on/Flpo-on-ChR2-EYFP) (Fig. 3a). This resulted in ChR2-labeling of neurons in the GPi as well as their axon terminals in LHb, without any labeling of LHA Vglut2 neurons (Fig. 3b, supplementary Fig. S3A-B). We first performed optogenetic stimulation of the Vglut2/Vgat^GPi-LHb^ neurons during a real-time place preference assay to determine their involvement in behavioral avoidance similarly to previous studies [13, 16]. In contrast to the predicted role for GPi-LHb projections in signaling aversion, we observed that optogenetic activation of Vglut2/Vgat^GPi-LHb^ neurons did not induce avoidance from the optogenetically stimulated compartment (Fig. 3c, supplementary video 1). The optogenetic activation of Vglut2/Vgat^GPi-LHb^ neurons did not induce any significant changes in speed (non-stimulated compartment 7.5 ± 1.4 cm/sec, stimulated compartment 7.3 ± 1.4 cm/sec, *P* > 0.1), grooming events (non-stimulated compartment 13 ± 1.7, stimulated compartment 11 ± 0.9, *P* > 0.1), or rearing events (non-stimulated compartment 41 ± 14.9, stimulated compartment 53 ± 13.1, *P* > 0.1). We confirmed that the optogenetic manipulation activated the ChR2 + Vglut2/Vgat^GPi-LHb^ neurons, quantified as the significant induction of the activity marker cFos (supplementary Fig. S5A-D). To further target the GPi-LHb pathway using additional genetic-viral strategies, we targeted Sst^GPi-LHb^ neurons with injections of a Cre-dependent retrograde AAV vector (rAAV DIO ChR2-mCherry) into the LHb of Sst-Cre mice (supplementary Fig. S5E-F, S6M-O), or through injections of a Cre-dependent AAV vector directly into the GPi of Sst-Cre mice (AAV DIO ChR2-mCherry) (supplementary Fig. S6D-F). In both cases, optogenetic activation of the Sst + GABA/glutamate GPi neurons failed to induce any significant aversive response (Fig. 3g). In addition, we performed small injections (70 nl) of AAV DIO ChR2-mCherry into the GPi of Vglut2-Cre mice (supplementary Fig. S6G-I), or optogenetically targeted all neurons in the GPi using a ubiquitous gene promoter to drive ChR2 expression (AAV CAG ChR2-mCherry) (supplementary Fig. S6J-L). Similarly, these animals did not show any significant avoidance to optogenetic activation of the GPi Vglut2 neurons (Fig. 3g). In summary, we were not able to induce aversive responses through optogenetic stimulation of the GPi-LHb pathway, based on either somatic or axonal stimulation using different viral and genetic targeting approaches (experimental details in supplementary Table 1). In contrast, when we specifically targeted ChR2 expression to LHA Vglut2 neurons through small stereotaxic viral injections (70 nl, AAV DIO ChR2-mCherry), we found that optogenetic stimulation of Vglut2^LHA-LHb^ neurons produced a significant and strong avoidance behavior (Fig. 3d–g, supplementary Fig. S6A-B, supplementary video 2). Furthermore, the avoidance response induced by optogenetic activation of the Vglut2^LHA-LHb^ neurons was frequency dependent (supplementary Fig. S6C), suggesting that this pathway can carry information about the value of aversive stimuli. From our data, we conclude that the LHb-projecting GPi neurons, which co-releases glutamate and GABA, do not mediate aversive signals. Instead, we found that aversive responses are mediated by the Vglut2^LHA-LHb^ population.

### The role of Vglut2^GPi-LHb^ and Vglut2^LHA-LHb^ neurons in action value

To further compare the role of the Vglut2/Vgat^GPi-LHb^ and Vglut2^LHA-LHb^ populations in shaping valence-based behaviors, we trained mice in an action value task [36]. In this probabilistic 2-choice switching task, mice chose freely between two nose poke ports, where poking into one port was rewarded (sucrose solution) with 75% probability, while the other port was non-rewarded. Since the rewarded port as well as the switching of the rewarded side (every 7–23 trials) were not indicated by any cues, mice must base their choices on the value of their previous actions (Fig. 3h, i). The task design allowed us to compare how the Vglut2/Vgat^GPi-LHb^ and Vglut2^LHA-LHb^ pathways influence the value of actions and updating the value of future choices. We performed optogenetic activation of the Vglut2^LHA-LHb^ neurons at the moment of outcome evaluation (10%, during reward port nose poke), and we found that it significantly biased mice to switch to the alternative port on the subsequent trial (Fig. 3j, l, n, supplementary Table 1). Interestingly, applying optogenetic stimulation of the GABA/glutamate co-releasing GPi neurons (Vglut2/Vgat^GPi-LHb^ neurons) had no significant effect on the action value (Fig. 3k, m, o), in contrast to the proposed role of the GPi-LHb in shaping action value [16]. These findings demonstrate that the Vglut2^LHA-LHb^ projection, and not the GPi GABA/glutamate co-releasing LHb projection, controls the outcome evaluation by encoding a negative value. These results further support the functional dichotomy in the encoding of negative value between the LHA-LHb and GPi-LHb pathways.

### Calcium imaging of LHA Vglut2 neurons during rewarding and aversive events

**Fig. 4:**
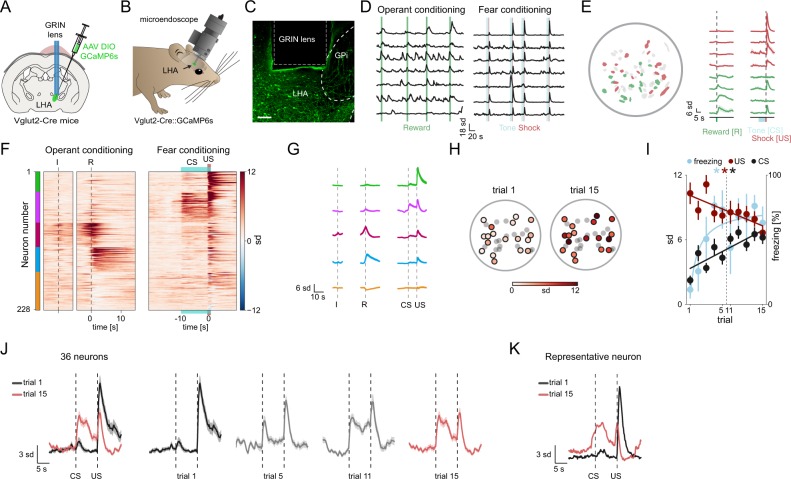
Activity of single LHA Vglut2 neurons in response to aversive and rewarding events. **a** Illustration of the experimental approach. To target GCaMP6s expression to LHA Vglut2 neurons, AAV DIO GCaMP6s (green) was injected into the LHA in Vglut2-Cre mice. GCaMP6s + Vglut2 neurons were imaged through a GRIN lens targeting the LHA. **b** Illustration of a mouse with miniaturized microscope. **c** Representative image showing Cre-dependent expression of GCaMP6s (green) in Vglut2 neurons and placement of GRIN lens above the LHA in a Vglut2-Cre mouse. **d** Representative calcium traces showing activity of individual LHA Vglut2 neurons from a single mouse in response to reward delivery (green lines) in operant conditioning, and to tone (blue, 10 sec) and mild foot shock (red, 1 sec) during fear conditioning. **e**: Left: Field of view map of individual LHA Vglut2 neurons color-coded according to response (red: shock responsive neurons, green: reward responsive neurons). Right: calcium traces showing the response of single imaged neurons. Green bar: reward delivery, blue bar: tone (CS), red bar: foot shock (US). **f** Clustering of individual LHA Vglut2 neurons based on their response to reward delivery in operant conditioning (left), and the CS and US during fear conditioning (right). Raster plot showing mean responses of individual LHA Vglut2 neurons during operant (left) and fear conditioning (right) recorded on the third day of conditioning. Neurons are sorted into five clusters indicated by the color bar on the left. Clusters: green *n* = 33 neurons, purple *n* = 51, red *n* = 40 neurons, blue *n* = 41 neurons, yellow *n* = 63 neurons. (I trial initiation by nose poke, R reward delivery upon food hopper entry, CS shock-predicting tone, US mild foot shock). **g** Average calcium trace for each cluster in **f** in response to initiation nose poke (**i**), reward (R), tone (CS) and foot shock (US) on third day of recordings (*n* = 228 neurons). **h** Field of view map of LHA Vglut2 neurons depicting the average calcium signal (sd) during the CS (first 5 sec), in the first (trial 1) versus fifteenth trial (trial 15). **i** The trial by trial average calcium signal of 36 CS-modulated LHA Vglut2 neurons (purple cluster in **f**) during the CS (first 5 sec after tone onset) and US (2 sec after shock onset). Red and black lines show the least-squares regression of the CS and US of the CS-modulated neurons. The trial by trial freezing response (light blue). Light blue line shows the least-squares regression of the freezing response on each trial transformed by the reciprocal function. **j** The average calcium trace of the 36 CS-modulated LHA Vglut2 neurons during presentation of the CS and US in the first trial (black trace) and the fifteenth trial (red trace). Gray traces: the US-to-CS response transfer shown in selected trials (trial 5, trial 11). **k** Calcium trace of a representative CS-modulated LHA Vglut2 neuron during presentation of the CS and US in the first trial (black trace) and the 15th trial (red trace). See supplementary Fig. S7 for details on behavioral schedule for imaging sessions and methods to extract calcium traces. See supplementary Fig. S8 for additional data on imaging of LHA Vglut2 neurons. Scale bars: 100 µm in **c**. Error bars and shading: mean ± s.e.m. *P < 0.001. All calcium imaging data analysis from 4 mice

To determine if the optogenetic manipulations reflected physiological neuronal activities of the Vglut2^LHA-LHb^ neurons during behavior, we further characterized their in vivo neuronal activity during positive and negative behavioral events. To first define the activity of all LHA Vglut2 neurons during positive and negative events, including neuronal representation of predictive stimuli, we performed in vivo imaging of the genetically encoded calcium sensor GCaMP6s [37] through a head-mounted microscope [38], during fear and reward conditioning behaviors (supplementary Fig. 7A). An AAV DIO vector (AAV DIO GCaMP6s) was injected into the LHA of Vglut2-Cre mice to express GCaMP6s in LHA Vglut2 neurons (Fig. 4a). We implanted GRIN lenses targeting the LHA, and imaged the real-time calcium dynamics with cellular resolution of a large number of Vglut2^LHA^ neurons (Fig. 4b, c) during reward conditioning in order to map the neural representation of rewards, and during exposure to mild foot shocks in fear conditioning to map aversive signals (Fig. 4d, e, supplementary Fig. S7B-I). To map the development of neural responses during learning, and to identify possible overlap in the representation of rewarding and aversive events or predictive cues, we imaged the activity of individual GCaMP6s + LHA neurons in response to rewards and shocks over several days. Non-negative matrix factorization and clustering of all the recorded calcium traces from 228 single LHA Vglut2 neurons was used to identify the main activity patterns that could differentiate the imaged neurons into candidate subpopulations (Fig. 4f, g). This analysis revealed five activity clusters that represented distinct responses in the reward task, or during learning over 3 days in the fear conditioning paradigm (supplementary Fig. S8A-B). We identified a cluster of neurons that was strongly activated by foot shocks, but showed limited response to rewards (Fig. 4g). Other clusters displayed instead a strong activation in response to rewards or upon entry to the rewarded port (Fig. 4g). When we analyzed the learning response in the fear conditioning paradigm, we found a group of neurons that over 15 trials developed an increase in the calcium signal during presentation of the tone, and these were uniformly distributed across the imaging field (Fig. 4h). Detailed mapping of individual neurons that were significantly modulated by the onset of the 10 s tone (conditioned stimulus, CS; purple cluster in Fig. 4g, supplementary video 3) predicting the upcoming event across fear conditioning trials revealed a subset of Vglut2^LHA^ neurons that over time displayed a linear increase in the response to the tone (CS), and a parallel linear decrease in the response to the shock (unconditioned stimulus, US), which also mirrored the behavioral response (freezing) (Fig. 4i–k). The response profile of the CS-modulated subset of LHA Vglut2 neurons, which first signals the aversive event (US, foot shock) and rapidly develops a predictive signal (CS), fulfills the criteria for an excitatory signal of negative value that can control the activity of LHb neurons.

### Calcium imaging of Vglut2^LHA-LHb^ neurons during rewarding and aversive events

**Fig. 5:**
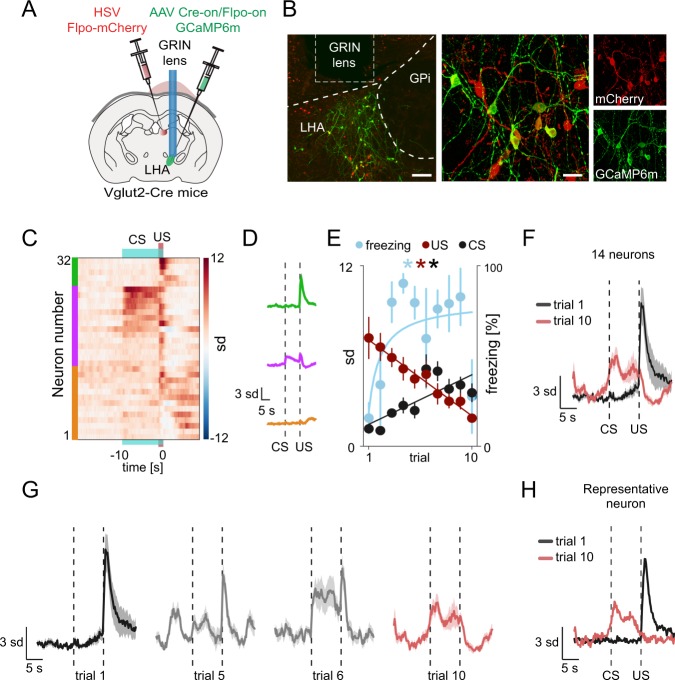
Vglut2^LHA-LHb^ neurons encode and predict aversive events. **a** Illustration of the experimental approach. GCaMP6m expression was restricted to LHb-projecting LHA Vglut2 neurons (Vglut2^LHA-LHb^) by injection of HSV Flpo-mCherry (red) into the LHb and of AAV Cre-on/Flpo-on GCaMP6m into the LHA in Vglut2-Cre mice. GCaMP6m + Vglut2^LHA-LHb^ neurons were imaged through a GRIN lens targeting the LHA. **b** Representative image showing expression of Flpo-mCherry (red) in retrogradely HSV-labeled LHA-LHb neurons, and GCaMP6m (green) only in Vglut2^LHA-LHb^ neurons. **c** Clustering of individual Vglut2^LHA-LHb^ neurons (*n* = 32 neurons, aligned between first and second day) showing the average calcium responses during fear conditioning (on second day). Clusters: green *n* = 4 neurons, purple *n* = 14 neurons, yellow *n* = 14 neurons. **d** Average calcium trace for each cluster in **e** in response to tone (CS) and foot shock (US) on the third day of recordings. **e** The average trial-by-trial, peak calcium responses of 14 CS-modulated Vglut2^LHA-LHb^ neurons (purple cluster in **c**) during the CS (first 5 s after tone onset) and US (2 s after shock onset). Red and black lines show the least-squares regression of the CS and US of the CS-modulated neurons. The trial by trial freezing response (light blue). Light blue line shows the least-squares regression of the freezing response on each trial transformed by the reciprocal function. **f** The trial by trial average calcium signal of 14 CS-modulated Vglut2^LHA-LHb^ neurons (purple cluster in **c**) during presentation of the CS and US in the first trial (black trace) and the tenth trial (red trace). **g** The average calcium trace of 14 CS-modulated Vglut2^LHA-LHb^ neurons (purple cluster in **c**) during presentation of the CS and US in the first trial (black trace) and the tenth trial (red trace). Gray traces: the US-to-CS response transfer shown in selected trials (trial 5, trial 6). **h** Calcium trace of a representative CS-modulated Vglut2^LHA-LHb^ neuron during presentation of the CS and US in the first trial (black trace) and the tenth trial (red trace). See supplementary Fig. S8 for data on reward responses, as well as dependence on motor behavior, of the CS-modulated Vglut2^LHA-LHb^ neurons. Scale bars: 100 µm in (B left), 20 µm in (B right). Error bars and shading: mean ± s.e.m. All calcium imaging data analysis from 3 mice

We next investigated whether specifically the Vglut2^LHA-LHb^ projecting neurons can represent and predict aversive, or rewarding, events. To target Vglut2^LHA-LHb^ exclusively, we designed an intersectional genetic-viral strategy to restrict expression of GCaMP6m to Vglut2^LHA-LHb^ neurons (Fig. 5a). Vglut2-Cre mice were injected into the LHb with a modified Herpes simplex virus optimized for retrograde labeling (HSV-Flpo-mCherry virus), as well as into the LHA with a Cre-on/Flpo-on-GCaMP6m AAV vector [39]. The experimental rationale was based on restricted expression of GCaMP6m in LHA neurons expressing Cre + (i.e., Vglut2 neurons) as well as Flpo + (i.e. projecting to LHb), thereby uniquely identifying the Vglut2^LHA-LHb^ neurons. This intersectional strategy resulted in GCaMP6m expression in Vglut2^LHA-LHb^ neurons and their axon terminals in LHb (Fig. 5b, supplementary Fig. S8C). We recorded as earlier, the calcium dynamics of GCaMP6-expressing Vglut2^LHA-LHb^ neurons during conditioning paradigms to map the neural representation of positive and negative value (supplementary Fig. S8D). To investigate how the aversive signal develops during learning of the CS-US association we analyzed activity of Vglut2^LHA-LHb^ neurons across the fear conditioning trials (Fig. 5c–h). Within a few trials of paired CS-US presentations, where the association between the CS and the US is expected to underlie fear learning and aversive event prediction, we found that Vglut2^LHA-LHb^ neurons showed over 10 trials a linear decrease in their calcium peak amplitude during the US and a corresponding linear increase during the CS as mice displayed a significant increase in the freezing behavior (Fig. 5e, f, supplementary video 4). Interestingly, the CS-modulated Vglut2^LHA-LHb^ neurons displayed a neural representation of a long-lasting conditioned response, since already the first CS exposure on the second day induced a sharp increase in the calcium signal (trial 6 in Fig. 5g, supplementary Fig. S8F). In addition, the activity of CS-modulated Vglut2^LHA-LHb^ neurons did not simply represent changes in motor behavior, as calcium signals were significantly higher during freezing behavior in CS periods (CS + ) compared to freezing occurring during intertrial intervals (supplementary Fig. S8F-G). Supporting the role of Vglut2^LHA-LHb^ neurons in encoding negative values, we found that these neurons did not encode pure sensory signals, as they were not activated by the first presentation of the 10 s auditory stimulus (Fig. 5f–h, supplementary material S8F), and they were also not activated by operant behavior or reward consumption (supplementary Fig. S8D).

In summary, the in vivo calcium imaging data show that Vglut2^LHA-LHb^ neurons can encode negative aversive events, and rapidly develop a CS-US association, forming the basis for a predictive signal of future aversive or negative events.

## Discussion

We have in this study presented evidence for the central role of a subtype of LHA Vglut2 neurons in mediating aversive signals through projections to LHb. To map the identity, localization, and physiology of the Vglut2^LHA-LHb^ neurons, and contrast that to the GABA/glutamate co-releasing GPi neurons, we first identified the distinct molecular signatures of Vglut2 neurons in the GPi and the adjacent LHA. Based on the distinct expression of Sst, Vglut2, and Vgat, we could genetically target the GABA/glutamate co-releasing GPi neurons that project to LHb. Connectivity and functional mapping supported that the Vglut2^GPi-LHb^ and Vglut2^LHA-LHb^ populations represent separate circuits, most likely with differential, and unique roles in action-selection and valence signaling. Interestingly, the Vglut2^LHA-LHb^ neurons receive a number of limbic inputs that have been associated with motivated and fear behaviors, including the preoptic area (MPO and LPO), bed nucleus of the stria terminalis (BST), amygdalar nuclei (sAMY), and periaqueductal gray (PAG). This connectivity profile motivated us to further investigate the role of the Vglut2^LHA-LHb^ population in aversive signals. We found a strong aversive response to optogenetic stimulation of the Vglut2^LHA-LHb^ neurons in the real-time place preference assay, and that this pathway can directly shape the value of actions in the probabilistic 2-choice switching task. Importantly, beyond the effects of optogenetic manipulation, we found that individual Vglut2^LHA-LHb^ neurons can encode an aversive event (e.g. mild foot shock), as well as rapidly develop a neural representation of the learned association between the tone and the upcoming shock during fear conditioning. Notably, our findings diverge from the predicted role of GABA/glutamate co-releasing GPi neurons in mediating aversive signals. Previous studies have targeted the rodent GPi using optogenetics and electrophysiology [13, 16], but without genetic access to differentiate between different neuron subtypes, or without the possibility to discriminate among subpopulations of Vglut2 neurons in the GPi/LHA border region. The anatomical distribution of several Vglut2 neuron types in the LHA/GPi border region requires more refined genetically restricted approaches than targeting of GPi neurons using VGlut2-Cre mice only. Optogenetic manipulation, or single unit recordings, of Vglut2 neurons in the GPi/LHA border region can potentially include Vglut2^LHA-LHb^ neurons when Vglut2 expression is used as the only criterion for identification.

To map the activity of LHA Vglut2 neurons during behavior, we imaged calcium signals from individual neurons during operant reward conditioning, where mice learn to perform actions resulting in the delivery of a reward, and during classical fear conditioning, where mice learn to associate specific sensory stimuli, (e.g., a tone) with the occurrence of an aversive event (e.g., a mild foot shock). Our in vivo calcium imaging data suggest a considerable functional diversity of the LHA Vglut2 population in the regulation of motivated behavior. Previous studies have identified that LHA neurons can respond to rewards as well as aversive events, and also to the conditioned stimulus [40–42]. More recently, LHA neurons have been shown to also shape the innate threat responses through their projections to LHb or periaqueductal gray [43, 44]. Whether those LHA neurons represent functionally and molecularly distinct neurons compared to the population we have mapped remains to be determined. Ultimately, the molecular diversity and spatial distribution of the different LHA neuron subtypes and how their circuit organization translates into functional specialization remains unknown. For example, LHA neurons responding to pleasant versus aversive taste stimuli can be found in the same area [45]. The LHA contains a large number of Vglut2 neurons, of which some map onto neuron subtypes defined by distinct neuropeptide expression patterns, whereas other populations are still of unknown identity and function [28]. A subpopulation of Vglut2 neurons defined by hypocretin (Hcrt) [46] expression has been shown to regulate reward seeking [47], but also awake-sleep states [48]. Optogenetic activation of GABAergic LHA neurons can induce self-stimulation behavior [49], and promote feeding [50], whereas LHA Vglut2 neurons that project to the VTA signal avoidance [51]. The signals representing positive reinforcement have been extensively studied primarily in basal ganglia circuits, whereas the studies of circuits responsible for instructing fear and aversive responses have focused on the amygdala circuitry. Neural representation of reward prediction signals has been found in dopaminergic neurons [52], and reward and punishment signals have been found in different types of midbrain neurons [53]. Although we did not observe strong modulation of Vglut2^LHA-LHb^ neurons by positive value (rewards), it is possible that the current technical limitations of calcium imaging preclude the detection of decreased activity in response to positive events. How the circuit organization of the LHA-LHb pathway and integration with downstream neuromodulatory systems as well as other fear circuits, ultimately together produce motivated behaviors and aversion remains to be determined. To better understand the differential contribution of the GPi versus LHA projections to LHb in guiding behavior, it will be important to determine how processing of discrete inputs is organized in LHb and what role different LHb output nuclei play in behavior. For example, GPi efferents target the lateral subdivisions of LHb [54], which in turn project to the rostral tegmental area (RMTg) [55], a pathway implicated in aversion [56]. In comparison, LHA projections also target medial parts of LHb, thereby potentially controlling other downstream targets, such as VTA, RMTg, or other brainstem nuclei [26]. Our results can be relevant for understanding circuit pathophysiology of anxiety disorders, which are thought to result from maladaptive fear consolidation or expression. We hypothesize that maladaptive synaptic plasticity in the Vglut2^LHA-LHb^ pathway, for example as a result of exposure to chronic stress or strong fear experiences, could play a critical role in the development of anxiety and mood disorders. By understanding the structure and function of the LHA-LHb circuitry and the synaptic mechanisms that underlie representation of fear and prediction of negative consequences, it will be possible to design intervention strategies for pathological fear conditions.

## Methods

### Mice

Experiments were conducted using adult male mice (25–30 g), either wild type C57BL/6 J (Charles River Laboratories), or transgenic mouse lines (Vglut2-Cre: Slc17a6^tm2(cre)Lowl^; Sst-Cre: Sst^tm2.1(cre)Zjh^, Vgat-Flpo: Slc32a1^tm1.1(flpo)Hze^, the Jackson Laboratory, USA), Cre-inducible TVA receptor [57], Cre-inducible H2bGFP reporter [58]. All transgenic mice used in experiments were heterozygous for the transgene. Mice were maintained under standard housing conditions with a 12-hour light cycle and with *ad libitum* access to food and water unless placed on a food restriction schedule. All food-deprived mice were restricted to 85–90% of their initial body weight by administering one feeding of 2.0–2.5 g of standard grain-based chow per day. All procedures were approved by the Swedish local ethical committee for animal experiments (Stockholms djurförsöksetiska nämnd, approval N166/15).

### Viral constructs

Purified and concentrated adeno-associated viruses (AAV) coding for Cre-inducible ChR2-mCherry (AAV5-EF1α-DIO-hChR2(H134R)-mCherry), GCaMP6s (AAV5-CAG-Flex-GCaMP6s) were packaged by the Penn Vector Core at University of Pennsylvania. Viruses coding for Cre and Flp-inducible GCaMP6m and ChR2-EYFP (AAV8-EF1α Con/Fon GCaMP6m or ChR2-EYFP) were produced in the laboratory of Dr. Karl Deisseroth (Stanford University).

The retrograde AAV AAV-EF1a-double floxed-hChR2(H134R)-EYFP-WPRE-HGHpA was a gift from Karl Deisseroth (Addgene viral prep # 20297-AAVrg; http://n2t.net/addgene:20298; RRID_Addgene_20298). The HSV-Flpo (HSV-hEF1a-mCherry-IRES-flpo) was purchased from the Viral Gene Transfer Core of the McGovern Institute for Brain Research at MIT. The helper virus TVA-V5-RG (AAV5-EF1a-DIO-TVA-V5-t2A-Rabies G) and Rabies-EGFP virus were cloned and produced in the Meletis laboratory.

### Viral injections and implants

Mice were anesthetized with isoflurane (2%) and placed into a stereotaxic frame (Harvard Apparatus, Holliston, MA). During the surgery the analgesic Buprenorphine (0.03 mg/kg) was administered subcutaneously (50 μl). The temperature of the mice was maintained at 36 °C with a feedback-controlled heating pad. For cell-type-specific retrograde tracing (Vglut2-Cre::TVA mice); a total volume of 0.3 μl of Rabies-EGFP virus [35] (3.03 × 10e9 particles/ml) was injected into the LHb (coordinates: AP −1.65 mm, ML .95 mm, DV −2.45 mm). For cell-type projection-specific monosynaptic retrograde tracing (Vglut2-Cre for LHA and GPi) a total volume of 0.07 μl (LHA or GPi injections) containing helper viruses TVA-V5-RG (AAV5-EF1a-DIO-TVA-V5-t2A-Rabies G) was injected into LHA (coordinates: AP −1.1 mm, L 1.1 mm, V −4.5 mm; 3 Vglut2-Cre mice), or into GPi (coordinates: AP −1.3 mm, L 1.7 mm, V −3.5 mm; 3 Vglut2-Cre) with a micropipette using a Quintessential Stereotaxic Injector (Stoelting, Wood Dale, IL). The pipette was held in place for 5 min after the injection before being slowly retracted from the brain. Post-injection analgesics were given (0.03 mg/kg Buprenorphine). After 21 days, 0.3 μl of Rabies-EGFP virus (3.03 × 10e9 particles/ml) was injected into the LHb (coordinates: AP −1.65 mm, L 0.95 mm, V −2.45 mm). For slice electrophysiology and optogenetics experiments, targeting and labeling of neuronal inputs was achieved by unilateral injection of 0.07 μl ChR2-mCherry (AAV5-EF1α-DIO-hChR2(H134R)-mCherry) (3 × 10e12 particles/ml) into the LHA (Vglut2-Cre mice *n* = 3, coordinates: AP −1.1 mm, L 1.1 mm, V −4.5 mm) in GPi (in Sst-Cre mice *n* = 3 or Vglut2-Cre mice *n* = 3, coordinates: AP −1.35 mm, L 1.75 mm, V −4.0 mm). For visualization of Sst neurons in GPi 0.3 μl AAV5-FLEX-tdTomato (4.8 × 10e12 particles/ml) was injected into the GPi as described above.

For behavioral experiments adult male Vglut2*-*Cre and Sst-Cre mice were bilaterally microinjected with 0.07 or 0.2 μl of ChR2-mCherry, respectively, into the LHA (coordinates: AP −1.1, mm L 1.1, V −4.5) and in the GPi (coordinates: AP −1.35, mm L 1.75, V −3.5). Vglut2-Cre/Vgat-Flpo were bilaterally microinjected with 0.2 μl of ChR2-EYFP Cre-on/Flpo-on in the GPi. For LHA and GPi terminal photostimulation experiments, mice were bilaterally implanted with optical fibers aimed directly above the LHb (coordinates: AP −1.65 mm, L 0.92 mm with 10° angle, and V −2.2 mm) or the cell bodies for the GPi (coordinates: AP −1.35 mm, L 1.75 mm, and V −3.5 mm).

For in vivo calcium imaging experiments mice received a unilateral 0.3 μl microinjection of AAV5-CAG-Flex-GCaMP6s into the LHA using the same stereotactic coordinates described above (AP −1.1 mm L 1.1 and V 4.5). For the projection specific in vivo calcium imaging experiments Vglut2-Cre mice received one injection of HSV-Flp (HSV-hEF1a-mCherry-IRES-flpo) in the LHb and a second in LHA with Cre and Flpo-inducible GCaMP6m (AAV8-EF1α Cre-on/Flpo-on GCaMP6m). Two weeks later, the microendoscope lens was implanted 100–200μm above the area of interest. For the procedure, after the craniotomy, portions of tissue above the area of interest were aspirated with a custom-made 0.5 mm diameter beveled syringe needle attached to a vacuum pump to create a clear entry point. The microendoscope lens (0.44 pitch, 0.47 NA, 0.5 mm in diameter, and 6.1 mm in length; Inscopix, Palo Alto, CA) was slowly lowered into tissue with a custom-made holder attached to a stereotactic arm and fixed with dental cement. Four to six weeks after the lens implantation animals were anesthetized placed in a stereotaxic frame, the miniaturized microscope with the baseplate attached was lowered until the field of view was in focus and the baseplate (Inscopix, Palo Alto, CA) was fixed with dental cement. Finally, a cover implant (Inscopix, Palo Alto, CA) was secured into the baseplate with a screw to protect the lens until imaging.

### Histology

Seven days after rabies virus injections, mice were deeply anaesthetized with pentobarbital and then perfused transcardially with 0.1 M PBS followed by 4% paraformaldehyde in PBS 0.1 M. Brains were removed and post fixed in 4% paraformaldehyde over night at 4 °C and then washed and stored at 0.1 M PBS. Coronal 60μm sections were cut using a vibratome (Leica VT1000, Leica Microsystems, Germany). Immunostaining was performed on free-floating sections in glass wells. Briefly sections were incubated for 1 h in 0.3% TritonX-100 in Tris–buffered saline (38 mM Tris-HCl, 8 mM Trizma base, 120 mM NaCl in extra pure water) and treated with a preheated (40°C) antigen retrieval solution (10 mM sodium citrate, 0,05% Tween20, pH:6) for 1–2 minutes. In order to block the non-specific antibody binding, sections were incubated in 5% Normal Donkey Serum in TBST (0,3% TritonX-100 in Tris–buffered saline), for 1 h at room temperature. Sections were subsequently incubated overnight at RT with primary antibodies followed by a 4 h incubation with the secondary antibodies. Primary antibodies used: guinea pig anti-parvalbumin (PV; 1:1000 dilution; Synaptic Systems; cat. No. 195 004); goat anti-SST D-20 (SST; 1:1000 dilution; Santa Cruz Biotechnology; sc-7819); chicken anti-V5 (V5; 1:500 dilution; Abcam; ab9113) rabbit anti-cFos (cFos; 1:500 dilution, Santa Cruz Biotechnology; sc-52). Fluorophore-conjugated secondary antibodies: staining was revealed the day after, when sections were washed twice for 10 min in TBST and incubated at room temperature for 2 h with the secondary antibodies (Alexa Fluor-488, Cy3 and Cy5 from Jackson ImmunoResearch Laboratories). For quantification of co-labeling of Rabies-EGFP and immunostaining, Z-stack and tiled images were captured on a Zeiss LSM 5 Pascal confocal laser-scanning microscope. Images were acquired using identical pinhole, gain, and laser settings for all brain regions. Identification of cell bodies and their anatomical position for each fluorescent channel were mapped to Openbrainmap (http://openbrainmap.org) and inputs per projection neurons for in Allen CCF 3.0 ontology were compiled using WholeBrain software [59]. Assessment of fiber placement was based on the lesion from the fiber in the tissue. Animals with unilateral viral expression or misplacement of optical fiber were excluded from the study.

### Single cell nuclear RNA sequencing

Vglut2-Cre mice were crossed with mice homozygous for Cre-dependent expression of histone 2B associated enhanced green fluorescent protein (H2B-EGFP) [60] to obtain a mouse line that expressed EGFP in the nuclei of Vglut2 + cells (Vglut2-H2BG mice). Animals were killed with an overdose of isoflurane and brains were rapidly extracted from the skull and submerged in ice-cold ACSF solution. The brains were then immediately sectioned (300μm) in ice-cold ACSF using a Leica vibratome (VT1200S, Leica). The 300 μm tissue slices were submerged in ice-cold Leibovitz’s L-15 medium (1 × , Gibco by life Technologies) in a petri dish and the lateral hypothalamic area (LHA) and the globus pallidus internal segment (GPi) were dissected out and stored in an Eppendorff tube (1 ml) containing ice-cold Leibovitz’s L-15 medium with 1 μl SUPERase RNase inhibitor (20 U/μl, ThermoFisher Scientific). The nuclei were isolated using a standard nuclear isolation protocol [61]. Briefly the tissue was homogenized in 2 ml lysis buffer using a Dounce Tissue Grinder (7 ml, VWR). 4 ml of 1.8 M sucrose solution was added and the homogenized solution was pipetted onto a sucrose cushion (2 ml) in a 10 ml Ultra-Clear centrifuge tube (BeckmanCoulter). Sample tubes were then spun at 26.500×*g* at 4 °C for 1.5 h. The supernatant was discarded and the nuclei pellet was resuspended in 500 μl Nuclear resuspension buffer and the mix was transferred to 5 ml FACS tubes. Single nuclei were isolated using Fluorescence-Activated Cell Sorting (FACS) and sorted into 384 well-plates containing 2.3 μl ice-cold lysis buffer. The plates containing the nuclei were immediately frozen on dry ice and stored on −80 °C until further processing. cDNA libraries were produced and sequenced using a Smart-seq2 protocol [62]. Sequencing of the single-nuclei libraries was performed using Illumina HiSeq 2000. The reads were mapped and aligned to mouse genome (mm10) and subsequently gene expression values were calculated as count values for each transcript. Analysis was performed on count values per nucleus. Only exons were included in the analysis. The sequencing data were analyzed using the Seurat package in R. The count data were log-scaled (log2), subsequently variance genes were identified by calculating their z-score of log(variance/mean). A linear dimensional reduction (PCA) was performed to obtain the genes that are differentially expressed throughout the population. Random sampling with 1000 replicates was done to determine the significant Principal Components in the dataset and a projected PCA was used to increase the gene list and to prevent losing potential marker genes. The resulting list of genes was again analyzed for principal components and randomly sampled (1000 replicates). The resulting significant principal components were implemented into a non-linear dimensional reduction (t-SNE) analysis. A subsequent density-based clustering was performed and markers per cluster were identified based on their differential expression. For details see R script in supplementary data.

### In situ hybridization

RNA in situ hybridization was performed using the RNAscope fluorescent multiplex assay (Advanced Cell Diagnostics, ACD, Hayward, CA) according to the manufacturer’s instructions. Briefly, mouse brains were perfused with 4% paraformaldehyde and left for post-fixation on 4%PFA overnight. The brains were subsequently cryoprotected by being immersed in a 15% sucrose solution (in PBS) overnight at 4 °C, the process was repeated with a 30% sucrose solution. The brains were then frozen in OCT on dry ice and stored at −80 °C. Cryosections were made using a cryostat (14 μm) and stored at −80 °C until further processing. Immediately before RNA in situ hybridization, cryosections were washed once in PBS (1 × ). Subsequently, sections were boiled in a pre-treatment reagent 2 for 5 min, washed in ddH2O and immersed in 100% ethanol. Sections were dried at room temperature and a hydrophobic barrier was drawn around the individual sections using an ImmEdge Hydrophobic Barrier Pen (Vector Labs, Inc.). All following incubation steps were performed in a HybEZ Hybridization System oven (ACD). Next, sections were incubated with pretreatment solution 4 (ACD) for 30 min at 40 **°**C. The sections were washed twice in fresh ddH2O and subsequently hybridized with multiplexed probes: Mm-Slc32a1 (channel 1), Mm-Slc17a6 (channel 2) and Mm-Sst (channel 3) for 2 h at 40 °C. After hybridization, the sections were washed twice in RNAscope wash buffer (ACD) and four consecutive amplification steps were performed using the RNAscope Fluorescent Multiplex Detection reagents 1–4 (amplifier 4 variant A was used in this case). After the last amplification step, the sections were immersed in DAPI for 30 s and immediately covered with a cover slip, using a protective fluorescent mounting medium. The sections were imaged under a Zeiss LSM 5 Pascal confocal laser-scanning microscope at ×40 magnification.

### Real-time place preference

Mice were placed in a custom-made two compartment behavioral arena separated by a wall with an opening in the middle (50 × 25 × 25 cm black plexiglass) for 20 min. The mouse performance was evaluated under three different conditions during three consecutive days. The first day optical fibers were connected to the animal but there was no light stimulation, the second day one of the compartments was paired with light stimulation (60 Hz, 1 ms pulse, 10 mW, 447 nm laser) and the third day stimulation paired side was switched. The speed (cm/s), discrete events of rearing (number of events of standing on the hind limbs), and discrete grooming events (number of events of mice in sitting position with licking of the fur, grooming with the forepaws, or scratching with any limb) where manually scored for the stimulated and non-stimulated compartments. Animal behavior was recorded with a CCD camera interfaced with a Biobserve software (Biobserve GmbH).

### Operant conditioning and fear conditioning

Mice with expression of GCaMP6s or GCaMP6m were implanted with a lens (GRIN lens), and then food restricted and trained in sound isolated operant conditioning chambers that were equipped with one nose poke port on one side and a food chamber on the other (Med Associates Inc.). The first nose-poke after the ITI (randomized average 40 sec) resulted in the onset of a tone as well as a house-light cue, which stayed on until the first entry into the food hopper and the delivery of a food pellet. Mice were trained until they reached 40 trials within a 45-minute session before starting the calcium imaging sessions. On the day of calcium imaging, the miniaturized microscope was attached on the magnetic plate and mice were allowed to habituate for 10 min. After the operant conditioning task, mice were directly moved (without removing the miniaturized microscope) into a sound isolated fear-conditioning chamber where they received five tones followed by mild foot-shocks (10 sec tone, 0.4 mA foot-shock the last 1 s of the tone) with randomized inter-shock intervals. Mice were recorded in the operant conditioning followed by fear conditioning for 3 consecutive days.

### Probabilistic 2-choice switching task

Mice were trained on a probabilistic 2-choice switching task [16, 36] in which the reinforcement outcomes of the two available choices were swapped intermittently and without warning. Under these circumstances, mice are known to adopt a “win-stay, lose-switch” strategy [16, 36], i.e. mice learn to prefer the most recently rewarded choice and to rapidly shift their efforts to the alternative option as soon as obtained outcomes fail to fulfill reward expectations. Hence analysis of choices made after optogenetic manipulation allows investigators to assess the optically targeted system’s impact on action selection [36] or outcome evaluation [16], depending on the timing of the light delivery. On each trial, mice chose between two identical nose poke ports fitted for sucrose reward delivery. Entry into one port had a 75 per cent chance of yielding a drop of sucrose, while a poke into the alternative port was non-reinforced. Every 7–23 rewarded trials, the two ports switched outcomes, the formerly reinforced port becoming non-reinforced and vice versa. The number of rewards necessary to trigger the next switch was randomly drawn at the start of every new block of trials delimited by such switches. As there was no cue indicating the “correct” port choice nor the occurrence of the switch on a given trial, mice had to rely solely on recently experienced outcomes in order to identify the choice most likely to produce a reward, prompting them to adopt the win-stay, lose-switch heuristic. The task was self-paced and required mice to begin every trial by actioning a central initiation port, located in between the two choice/reward ports. LED lights within the center and peripheral ports cued mice to perform trial initiation or left/right choice, respectively. Port entries were detected by infrared photodiode/phototransistor pairs placed inside each port. Optogenetic stimulation (500 ms duration, 30 Hz, 5 ms pulse, 10 mW, 447 nm laser) was delivered time-locked to rewarded port entry randomly on 10% of the trials [16]. Our aim was to weight and contrast the effects of reinforcement outcomes (reward, no reward) and outcome-time optogenetic stimulation on subsequent choices. We estimated these effects, for each animal individually, using the published logistic regression model [16, 36].

### Electrophysiology

Animals were anesthetized with isoflurane and decapitated. Brains were removed and immediately placed into ice cold cutting solution (2.5 mM KCl, 1.25 mM NaH2PO4, 25 mM NaHCO3, 0.5 mM CaCl2, 7 mM MgCl2, 10 mM Dextrose, 205 mM sucrose and gassed with 95%O2/5%CO2). Brains were sliced coronally on a Vibratome 1200VT (250–300 μm) throughout the anterior-posterior axis of the habenula. Slices were immediately transferred to a slice chamber containing aCSF recording solution (125 mM NaCl, 2.5 mM KCl, 1.25 mM NaH2PO4, 25 mM NaHCO3, 2 mM CaCl2, 1 mM MgCl2, 25 mM Dextrose, and continually bubbled with 95%O2/5%CO2) maintained at 34 °C and allowed to rest for 30 min. The slice chamber was allowed to return to room temperature for an additional 30 min rest prior to recording.

Recordings were made in the visualized terminal fields of projections into the LHb. All experiments were carried out in current clamp mode. Recording setup consisted of a HEKA EPC9/2 with internal DAC board, on a Leica DMLFSA microscope with a Hamamatsu ORCA-05G digital camera. Traces were filtered at 2 KHz. Data was acquired with the Patchmaster software (HEKA Elektronic) and analyzed using Matlab (The Mathworks Inc). Visualization of fluorescence in slices was achieved with an EQB 100 mercury lamp and filter cubes. Light stimulation was elicited with a blue (470 nm) LED (420 lm @ 350 mA Luxeon Star LED) with a 700 mA BuckPuck DC driver and delivered through the microscope eyepiece. Light intensity measured on the tissue slice ranged from 0.2–2.2 mW and pulse length ranged from 0.2–5 ms. Pipettes of 7–9MΩ were pulled on a P-1000 Flaming/Brown micropipette puller (Sutter Instruments) using borosilicate glass. To enhance GABAergic inhibitory components that contribute to synaptic transmission, a low-chloride internal solution was used (130mM K-gluconate, 5 mM KCl, 10 mM HEPES, 10 mM Na2-phosphocreatine, 4 mM ATP-Mg, 0.3 mM GTP-Na). Upon break-in to whole-cell configuration, test light pulses were applied to confirm connectivity. LHb neurons responding to efferent stimulation were then characterized by their firing patterns followed by the experiments designed to measure efferent synaptic properties. The monosynaptic nature of postsynaptic potentials as a response to optogenetic stimulation of LHA Vglut2 + and GPi Sst + projections were ensured by bath application of TTX (1 µM; Tocris) and 4-AP (100 µM; Sigma-Aldrich). During optogenetic stimulation protocols in all experiments the membrane potential was depolarized (−30mV) and held above the reversal potential of Cl (Ecl = −78mV) generating a GABA drive of approximately 50 mV. GABA-A receptor antagonist gabazine (10 µM; Sigma-Aldrich) was introduced to the recording chamber upon encountering biphasic responses or NBQX (10 mM; Tocris) to demonstrate whether a hyperpolarizing component could be revealed after abolishing the excitatory component of the postsynaptic response. The synaptic properties of LHA Vglut2 + and GPi SST + projections onto LHb neurons were probed using multiple light pulse train protocols.

### Calcium imaging analysis

Raw calcium imaging videos (1440 × 1080, 20 fps) were preprocessed, motion corrected, and down sampled (4 × 4 pixels spatial binning, 5 fps) using the Mosaic software (Inscopix). The processed recordings were exported as TIFF files. We manually defined region of interest (ROI) masks for individual neurons using ImageJ’s ROI Manager. To aid ROI definition, we computed normalized videos of relative changes in fluorescence over time; df(t)/f0 = (f(t) – f0)/f0, where f0 denotes the median projection image of the whole movie. ROIs were then drawn on grouped maximum intensity projections (subsets of 50 frames). Next, local neuropil-corrected fluorescence traces were calculated for each ROI: f(corrected) = f(ROI)-f(neuropil). Local neuropil fluorescence transients were derived from 10-pixel-wide annuli encircling their corresponding ROI. Pixels overlapping with ROIs were removed from ROI and neuropil masks prior to signal extraction. A baseline was fit to each trace in the form of a rolling 10th percentile filter (window width 30 s) and subtracted. Finally, each trace was re-expressed in terms of standard deviation (sd). When calculating the sd, we excluded calcium transients of persistent (>1 s) elevations above 10% of the trace’s fluorescence maximum. To quantify calcium traces based on df/f instead of sd, we used a published approach [63].

To categorize Vglut2 LHA neurons into response types, we first computed the average response of each neuron, time-locked to: (i) tone onset and shock, and (ii) trial initiation by nose poke, and (iii) reward delivery. To categorize Vglut2 LHA-LHb neurons into response types, we computed the average response of each neuron, time-locked to tone onset and shock. The average response of each neuron was down sampled 5 times by averaging (in 1-second bins) and concatenated into one session-average response profile. We then applied non-negative matrix factorization (NMF, Nimfa, http://stanford.edu/~marinka/nimfa/) to cluster the response profiles. Responses from different days were treated independently. The NMF algorithm was initialized using the Random Vcol method, minimized the Kullback-Leibler divergence, and was run 300 times to achieve a stable consensus clustering [64]. Pairs of neurons were clustered together based on the proportion (0–1) of factorization runs that grouped them together (the consensus). We manually selected the factorization rank (i.e. the target number of clusters) that maximized the robustness and homogeneity of the resulting clusters.

To investigate whether the CS response was learned, we computed trial-by-trial tone and shock responses for neurons in the CS-modulated cluster in the final recording session. The ROI representing each individual neuron was manually aligned between days. Tone and shock responses were defined as the average fluorescence during the first 5 s following tone onset and the first 2 s following shock onset. We performed least-squares regression on these responses, using trial number as the independent variable. Freezing responses were regressed on trial number (transformed by the reciprocal function). All custom-written image processing, analysis, and visualization code was written in the Python programming language.

## Supplementary information

Legends for supplementary material

supplementary table 1

supplementary video 1

supplementary video 2

supplementary video 3

supplementary video 4

supplementary figure 1

supplementary figure 2

supplementary figure 3

supplementary figure 4

supplementary figure 5

supplementary figure 6

supplementary figure 7

supplementary figure 8
