# Supplementary material for: A hypothalamus-habenula circuit controls aversion
Source: Mol Psychiatry. 2019 Feb 12;24(9):1351–68. doi: 10.1038/s41380-019-0369-5 (PMC6756229; doi:10.1038/s41380-019-0369-5)
Supplement: Supplementary file 1 — Legends for supplementary material [file 41380_2019_369_MOESM1_ESM.docx]

**LEGENDS FOR SUPPLEMENTARY MATERIAL**

**S1. Whole brain mapping of Vglut2-expressing neurons that project to LHb.**

(**A**) Coronal brain sections from a Vglut2-Cre:TVA mouse injected with EnvA-coated Rabies-EGFP into the LHb (see Fig. 1A-D). Vglut2-expressing (Vglut2) neurons projecting to the LHb (white) are found throughout the anteroposterior extent of the brain.

(**B**) Examples of Rb-EGFP-labeled Vglut2 neurons (green) in main input regions.

Orientation of coronal images: medial (M), dorsal (D).

Abbreviations: Anterior amygdalar area (AAA), Nucleus accumbens (ACB), anterior commissure, olfactory limb (aco), anterior commissure, temporal limb (act), Anterior hypothalamic nucleus (AHN), Bed nuclei of the stria terminalis, anterior division, anterolateral area (al), Bed nuclei of the stria terminalis, anterior division, anteromedial area (am), Bed nuclei of the stria terminalis (BST), Caudoputamen (CP), Central amygdalar nucleus (CEA), Cortical amygdalar area, anterior part (COAa), Dorsal nucleus raphe (DR), fornix (f), Fundus of striatum (FS), Globus pallidus, internal segment (GPi), internal capsule (ic), Lateral hypothalamic area (LHA), Lateral preoptic area (LPO), Medial amygdalar nucleus, anterodorsal part (MEAad), Medial preoptic area (MPO), Diagonal band nucleus (NDB), Nucleus of the lateral olfactory tract (NLOT), superior cerebelar peduncles (scp), Substantia innominata (SI), Periaqueductal gray (PAG).

Scale bars: 1 mm in (A), 200 µm in (B).

**S2. Definition of the GPi-LHA border region.**

(**A**) Left: In situ hybridization for mapping the expression of Vgat, Vglut2 and Sst in single neurons in the GPi-LHA border region (left hemisphere). Middle: depiction of segmentation of Vglut2+/Sst-/Vgat- (red), Vglut2-/Sst+/Vgat+ (green) and Vglut2+/Vgat+/Sst+ (purple) neurons. Right: The GPi and LHA border can be defined based on the differential distribution of neurons with distinct gene expression. The Vglut2+/Sst+/Vgat+ (purple) neurons are found in the GPi, while Vglut2-/Sst+/Vgat+ neurons are found in the LHA. Vglut2+/Sst-/Vgat- neurons are enriched in the LHA.

(**B**) In situ mapping of the anatomical distribution of Sst+/Vgat+/Vglut2+ co-expressing neurons (Sst/Vgat/Vglut2; purple), Vglut2+/Vgat-/Sst- neurons (Vglut2; red), and Vglut2-/Sst+/Vgat+ co-expressing neurons (Sst/Vgat; green) in relation to the GPi-LHB borders in four different anteroposterior coronal planes. From left to right: n = 194, n = 444, n = 296, n = 84 mapped neurons.

(**C**) ISH data from the Allen Brain Atlas depicting in situ hybridization of the expression of Parvalbumin (Pvalb, left) and Somatostatin (Sst, right). The cyan and orange delineations indicate the location of Pvalb-expressing and Sst-expressing neurons, respectively, within the GPi.

(**D**) Coronal brain section showing ChR2-EYFP expression in Vglut2+/Vgat+ neurons in the GPi in a Vglut2-Cre/Vgat-Flpo mice injected with AAV Cre-on/Flpo-on ChR2-EYFP into the GPi. Orange delineation indicates the location of Vglut2+/Vgat+ cell bodies in the GPi.

(**E**) Representative image of coronal brain section of GPi showing expression of Pvalb, Sst and tdTomato in a Vglut2-Cre mouse injected with AAV DIO tdTomato into the GPi-LHA border region.

(**F**) Mapping of the anatomical distribution of Vglut2+/Sst+/Pvalb- (Vlut2/Sst; red, n = 480), Vglut2+/Sst-/Pvalb- (Vglut2; green, n = 696) and Vglut2-/Sst-/Pvalb+ (Pvalb; blue, n = 328) neurons in relation to the GPi-LHA border. Vglut2 neurons mapped by tdTomato expression. Cyan delineation: defines the GPi motor pathway (Pvalb+ neurons). Orange delineation: defines the GPi to LHb pathway (Vglut2+/Vgat+/Sst+ neurons). Data from 8 superimposed sections along the anteroposterior axis from one animal.

(**G**) Illustration of the distribution of Vglut2+/Sst+/Pvalb- (Vglut2/Sst; red), Vglut2+/Sst-/Pvalb- (Vglut2; green) and Vglut2-/Sst-/Pvalb+ (Pvalb; blue) neurons along the anteroposterior extent of the GPi (distance from bregma -0.94 to -1.58 mm) (mapped in (F)).

(**H**) 4 of the 8 coronal sections used to generate the map in (F) and (G).

Scale bars: 200 µm in (A-D and F-H), 20 µm in (E).

**S3. Genetic targeting and anatomical distribution of Vglut2/Vgat^GPi-LHb^ and Sst^GPi-LHb^ neurons.**

(**A**) Representative images of coronal brain sections (left hemisphere). Left: injection of AAV Cre-on/Flpo-on-ChR2-EYFP into the GPi of Vglut2-Cre/Vgat-Flpo mice restricts ChR2-EYFP expression (green) to Vglut2+/Vgat+ cell bodies in the GPi. Middle: close-up of ChR2-EYFP labelled Vglut2/Vgat neurons in the GPi. Right: The axon terminals of ChR2-EYFP labelled Vlut2+/Vgat+ neurons are restricted to the lateral part of the LHb.

(**B**) Coronal brain sections from a Vglut2-Cre/Vgat-Flpo mouse injected with AAV Cre-on/Flpo-on-ChR2-EYFP into the GPi. Note the restricted ChR2-EYFP expression (white) in GPi neurons, and the specificity in the axon termination zone in the LHb. The GPi-LHb fiber tract is visible along the entire anteroposterior extent.

(**C**) Representative images of coronal brain sections (left hemisphere). Left: injection of the retrogradely transported rAAV DIO ChR2-mCherry into the LHb of Sst-Cre mice restricts ChR2-mCherry expression (red) to Sst+ cell bodies in the GPi. Middle: close-up of ChR2-mCherry labelled GPi Sst neurons. Right: The axon terminals of ChR2-mCherry labelled GPi Sst neurons are restricted to the lateral part of the LHb.

(**D**) Representative images of coronal brain sections showing the absence of ChR2-mCherry (red) labelled LHb-projecting Sst+ neurons in LHA and adjacent regions for the experiment shown in (C).

(**E**) Representative images of coronal brain sections from a Sst-Cre mouse injected with the retrogradely transported rAAV DIO ChR2-mCherry into the LHb. Note the restricted ChR2-mCherry expression (white) in GPi neurons, and the specificity in the axon termination zone in the LHb. The GPi-LHb fiber tract is visible along the entire anteroposterior extent.

Abbreviations: anterior commissure, olfactory limb (aco), Globus pallidus, internal segment (GPi), Lateral hypothalamic area (LHA), internal capsule (ic), Lateral habenula (LHb), Medial habenula (MHb), nigrostriatal tract (ns), stria medullaris (sm), fornix (f).

Scale bars: 100 µm in (A and C left and right images), 20 µm in (A and C middle image), 500 µm in (D), 1mm in (B and E).

**S4. Whole brain mapping of inputs to Vglut2^GPi-LHb^ and Vglut2^LHA-LHb^ projecting neurons.**

(**A**-**C**) AAV DIO TVA-V5 was injected into the GPi or the LHA in Vglut2-Cre mice. Rb-EGFP was injected into the LHb in all animals, thereby tracing of the input system of Vglut2^GPi-LHb^ or Vglut2^LHA-LHb^ projecting neurons.

(**A**) Mapping of the location of starter neurons (co-expressing TVA-V5 and Rb-EGFP) in the mouse brain reference atlas in individual animals. Vglut2^GPi-LHb^: n = 3 mice, n = 96 starter neurons (blue), Vglut2^LHA-LHb^ n = 3 mice, n = 163 starter neurons (red). Data shown from 5 superimposed coronal sections per animal.

­­­­(**B**) Examples of Rb-EGFP labeled (green) presynaptic neurons in key areas giving input to Vglut2^LHA-LHb^ neurons, including the BST, CEA, LPO, VTA, SNc, DR and PAG.

(**C**) Details from the mapping of Rb-EGFP labeled input neurons giving input to Vglut2^GPi-LHb^ (blue; data from one representative animal), and Vglut2^LHA-LHb^ neurons (red; data from one representative animal), respectively, detected in the BST (left), CEA (middle), and PAG (right).

Orientation of coronal images: lateral (L), dorsal (D).

Abbreviations: anterior commissure, olfactory limb (aco), Bed nuclei of the stria terminalis, anterior division, anterolateral area (al), Bed nuclei of the stria terminalis, anterior division, anteromedial area (am), Basolateral amygdalar nucleus (BLA), Bed nuclei of the stria terminalis (BST), Central amygdalar nucleus, lateral part (CEAl), Central amygdalar nucleus, medial part (CEAm), Caudoputamen (CP), Central amygdalar nucleus (CEA), Dorsal nucleus raphe (DR), Globus pallidus, external segment (GPe), Globus pallidus, internal segment (GPi), Lateral hypothalamic area (LHA), Lateral preoptic area (LPO), Lateral septal nucleus, ventral part (LSv), Bed nuclei of the stria terminalis, anterior division, oval nucleus (ov), Periaqueductal gray (PAG), Striatum-like amygdalar nuclei (sAMY), Substantia nigra, compact part (SNc), Substantia innominata (SI), Substantia nigra, reticular part (SNr), Ventral tegmental area (VTA), Zona incerta (Zi).

Scale bars: 1 mm in (A), 200 µm in (B and C).

**S5. Confirmation of optogenetic activation of Vglut2/Vgat^GPi-LHb^ neurons or Sst^GPi-LHb^ neurons.**

(**A**-**D**) To specifically target ChR2 to GABA/glutamate co-releasing GPi neurons, double transgenic Vglut2-Cre/Vgat-Flpo mice (n = 4) were bilaterally injected with AAV Cre-on/Flpo-on-ChR2-EYFP into the GPi (Fig. 3A). This resulted in ChR2-labeling of GPi neurons and their axon terminals in LHb (Fig. 3B). Bilateral optogenetic stimulation was targeted to the GPi.

(**A**) Mice were placed in a small chamber for 60 min (habituation). The Vglut2/Vgat^GPi-LHb^ neurons were thereafter optogenetically stimulated (447 nm, 10 mW) for 40 min (30 Hz, 5 ms pulse, 500 ms on / 500 ms off). Mice were perfused 50 minutes later.

(**B**) Representative images showing ChR2-EYFP (green) and cFos (red) expression in the GPi of the non-stimulated (left) and stimulated (right) hemisphere.

(**C**) Close-up of Vglut2/Vgat^GPi-LHb^ neurons co-expressing cFos (red), Sst (blue), and ChR2-EYFP (green) in the GPi of the stimulated hemisphere.

(**D**) Quantification of the number of GPi Sst neurons with ChR2-EYFP expression (Sst+ and EYFP+) that displayed high levels of cFos (cFos^High^: red) in the non-stimulated hemisphere (off; 84/596 neurons) versus stimulated hemisphere (on: 395/484 neurons) (n = 4 mice).

(**E**) Illustration of the experimental approach. The retrogradely transported rAAV DIO ChR2-Cherry was bilaterally injected into the LHb of Sst-Cre mice to specifically target ChR2 to Sst-expressing GPi neurons that project to the LHb (Sst+^GPi-LHb^ neurons). This resulted in ChR2-labeling of neurons in the GPi as well as their axon terminals in LHb. Bilateral optogenetic stimulation was targeted to the GPi. Red box: location of images in left and middle panels in (F). Blue box; location of images in right panel in (F).

(**F**) Representative images, right hemisphere. Left: ChR2-mCherry expression (red) in Sst^GPi-LHb^ neurons projecting to the LHb. Middle: ChR2-mCherry expression (red) is limited to neurons in the GPi. Right: ChR2-mCherry expression (red) in LHb from labeling of the axon terminals of Sst^GPi-LHb^ neurons.

(**G**) Mice were placed in a small chamber for 60 min (habituation). The Sst^GPi-LHb^ neurons were thereafter optogenetically stimulated (447 nm, 10 mW) for 40 min (30 Hz, 5 ms pulse, 500 ms on / 500 ms off). Mice were perfused 50 minutes later.

(**H**) Representative images showing ChR2-mCherry (red) and cFos (green) expression in the GPi of the non-stimulated (left) and stimulated (right) hemisphere.

(**I**) Close-up of Sst^GPi-LHb^ neurons co-expressing cFos (green), Sst (blue), and ChR2-mCherry (red) in the GPi of the stimulated hemisphere.

(**J**) Quantification of the number of GPi Sst neurons with ChR2-mCherry expression (Sst+ and mCherry+) that displayed high levels of cFos (cFos^High^: green) in the non-stimulated hemisphere (off; 53/487 neurons) and stimulated hemisphere (on: 317/406 neurons) (n = 4 mice).

Scale bars: 100 µm in (B, F and H), 20 µm in (C and I).

**S6. Strategies for optogenetic manipulation of GPi and LHA LHb-projecting neurons**.

(**A**) Illustration of the experimental approach. AAV DIO ChR2-mCherry (red) was bilaterally injected into the LHA of Vglut2-Cre mice to express ChR2 in LHA Vglut2 neurons. Optogenetic stimulation was targeted to ChR2-expressing Vglut2^LHA-LHb^ axon terminals in the LHb. Red boxes: location of lower panels in (B). Blue boxes; location of upper panels in (B).

(**B**) Representative images of coronal brain sections. Upper panels: ChR2-mCherry-expression in Vglut2^LHA-LHb^ axon terminals in the LHb. Lower panels: ChR2-mCherry-expression in Vglut2^LHA-LHb^ cell bodies in the LHA.

(**C**) Quantification of the frequency-dependent aversive response generated by the optogenetic stimulation of the axon terminals of Vglut2^LHA-LHb^ neurons (red data points; Vglut2-Cre mice, n = 4 mice). The behavioral response of control mice (green data points; wildtype mice, n = 6) injected with AAV CAG-EYFP into the LHA to 60 Hz optogenetic stimulation of axon terminals in LHb. Dashed lines show s.d.

(**D**) Illustration of the experimental approach. AAV DIO ChR2-mCherry (red) was bilaterally injected into the GPi of Sst-Cre mice (n = 22 mice) to express ChR2 in GPi Sst neurons. Optogenetic stimulation was targeted to ChR2-expressing axon terminals in the LHb. Red box: location of left panel in (E). Blue box; location of right panel in (E).

(**E**) Representative images of coronal brain sections. Left: ChR2-mCherry expression (red) in Sst+ cell bodies in the GPi. Right: ChR2-mCherry expression in the axon terminals of GPi Sst neurons projecting to LHb.

(**F**) Optogenetic stimulation in the place preference assay. Representative trace of the locomotion of a Sst-Cre mouse expressing ChR2-mCherry in GPi Sst neurons. Blue light (447 nm) stimulation (60 Hz, 1 ms pulses) of the ChR2+ axon terminals in the LHb did not induce a place aversion.

(**G**) Illustration of the experimental approach. AAV DIO ChR2-mCherry (red) was bilaterally injected into the GPi of Vglut2-Cre mice (n = 7 mice) to express ChR2 in GPi Vglut2 neurons. Optogenetic stimulation was targeted to ChR2-expressing Vglut2^GPi-LHb^ axon terminals in the LHb. Red box: location of left panel in (H). Blue box; location of right panel in (H).

(**H**) Representative images of coronal brain sections. Left: ChR2-mCherry expression (red) in Vglut2^GPi-LHb^ bodies in the GPi. Right: ChR2-mCherry expression in the axon terminals of Vglut2^GPi-LHb^ neurons projecting to LHb.

(**I**) Optogenetic stimulation in the place preference assay. Representative trace of the locomotion of a Vglut2-Cre mouse expressing ChR2-mCherry in GPi Vglut2 neurons. Blue light (447 nm) stimulation (60 Hz, 1 ms pulses) of the ChR2+ axon terminals in the LHb did not induce a place aversion.

(**J**) Illustration of the experimental approach. AAV CAG ChR2-mCherry (red) was bilaterally injected into the GPi of wildtype mice (n = 7 mice) to express ChR2 in GPi neurons. Optogenetic stimulation was targeted to ChR2-expressing GPi axon terminals in the LHb. Red box: location of left panel in (K). Blue box; location of right panel in (K).

(**K**) Representative images of coronal brain sections. Left: ChR2-mCherry expression (red) in GPi cell bodies. Right: ChR2-mCherry expression in the axon terminals of neurons projecting to LHb.

(**L**) Optogenetic stimulation in the place preference assay. Representative trace of the locomotion of a wildtype mice mouse expressing ChR2-mCherry in GPi neurons. Blue light (447 nm) stimulation (60 Hz, 1 ms pulses) of the ChR2+ axon terminals in the LHb did not induce a place aversion.

(**M**) Illustration of the experimental approach. Retrograde rAAV DIO ChR2-mCherry (red) was bilaterally injected into the LHb of Sst-Cre mice (n = 6 mice) to express ChR2 in GPi Sst neurons. Optogenetic stimulation was targeted to ChR2-expressing Sst+ cell bodies in the GPi. Red box: location of left panel in (N). Blue box; location of right panel in (N).

(**N**) Representative images of coronal brain sections. Left: ChR2-mCherry expression (red) in Sst+ cell bodies in the GPi. Right: ChR2-mCherry expression in the axon terminals of Sst^GPi-LHb^ neurons projecting to LHb.

(**O**) Optogenetic stimulation in the place preference assay. Representative trace of the locomotion of a Sst-Cre mouse expressing ChR2-mCherry in GPi Sst neurons. Blue light (447 nm) stimulation (60 Hz, 1 ms pulses) of the ChR2+ cell bodies in the GPi did not induce a place aversion.

All scale bars = 100 µm.

**S7. Approach for extracting calcium signals from individual neurons after in vivo calcium imaging.**

(**A**) Outline of the training and testing schedule for operant conditioning and fear conditioning tasks for the calcium imaging experiments.

(**B-E**) Procedure to compute local background-corrected calcium signal expressed as standard deviations of baseline noise.

(**B**) To correct the raw fluorescence time series of a given ROI (f(ROI), blue trace and blue mask) for background fluorescence, we subtracted the signal measured in the surrounding neuropil (f(neuropil), grey trace and grey mask).

(**C**) We then estimated the local baseline (orange line) of the background-subtracted trace to be the 10th percentile of the intensity values within a +/- 15 second wide sliding-window (grey shading and histogram) and deducted it.

(**D**) The distribution of baseline noise was obtained over the entire trace (grey shading and histogram), excluding all frames pertaining to calcium peaks, and its standard deviation calculated (green shading). Peaks were defined as periods within which fluorescence exceeded a cut-off of 10% of the trace's maximum for a second or longer (identified using a 1 second sliding minimum, orange line).

(**E**) Raw fluorescence change (df) from baseline divided by the standard deviation (sd) of the baseline noise.

(**F-H**) Example of alternative quantification of calcium traces (based on df/f) for the imaging data of LHA Vglut2 neurons. This is directly comparable to the quantification shown in Fig. 4.

(**F**) Raster plot based on df/f to plot calcium signal, showing mean responses of individual LHA Vglut2 neurons during operant (left) and fear conditioning (right) recorded on the third day of conditioning. Neurons are sorted into five clusters indicated by the color bar on the left. (I: trial initiation by nose poke; R: reward delivery upon food hopper entry; CS: shock-predicting tone; US: mild foot shock).

(**G**) Average calcium trace (using df/f to plot calcium signal) for each cluster in (F) in response to initiation nose poke (I), reward (R), tone (CS) and foot shock (US) on third day of recordings (n = 228 neurons).

(**H**) The trial by trial average calcium signal (using df/f to plot calcium signal) of the CS-modulated LHA Vglut2 neurons (purple cluster in (F)) during the CS (first 5 sec after tone onset) and US (2 sec after shock onset). Red and black lines show the least-squares regression of the CS and US of the CS-modulated neurons. The trial by trial freezing response (light blue). Light blue line shows the least-squares regression of the freezing response on each trial transformed by the reciprocal function.

(**I**) Illustration of approach to align neurons between days. ROIs from different days were overlaid and the neurons were mapped based on their overlap and their relative position with the surrounding ROIs.

Error bars and shading: mean ± s.e.m. * *P* < 0.001. All calcium imaging data analysis from 4 mice.

**S8. Vglut2^LHA-LHb^ neurons encode and predict aversive events.**

(**A**) Clustering of individual LHA Vglut2 neurons based on their response to reward delivery in operant conditioning (left) and the CS and US during fear conditioning (right) on day 2 of recording.

(**B**) Raster plots showing response of individual LHA Vglut2 neurons on day 1 and day 3 of recording. Clustering is based on day 3 response to reward delivery in operant conditioning (left) and the CS and US during fear conditioning (right). Neurons are sorted in the same order in both raster plots, since neurons have been aligned between day 1 and day 3.

(**C**) Top: Left: GCaMP6m expression was restricted to LHb-projecting LHA Vglut2 neurons (Vglut2^LHA-LHb^) by injection of HSV Flpo-mCherry (red) into the LHb followed by injection of AAV Cre-on/Flpo-on-GCaMP6m into the LHA in Vglut2-Cre mice. GCaMP6m+ Vglut2^LHA-LHb^ neurons were imaged through a GRIN lens targeting the LHA. Top left: overview representative image showing GCaMP6m+ Vglut2^LHA-LHb^ neurons. Top right: magnification of area shown in boxed area (LHA) in left panel. Bottom left: co-localization of mCherry (red) and GCaMP6m (green) expression in Vglut2^LHA-LHb^ neurons. Bottom right: magnification of boxed area in top left (LHb) showing GCaMP6m-expressing Vglut2^LHA-LHb^ axon terminals in the LHb.

(**D**) Raster plot depicting the average calcium responses of Vglut2^LHA-LHb^ neurons (n = 62 neurons from 3 mice) to trial initiation, and reward delivery in operant conditioning (left), and the tone and shock during fear conditioning (right).

(**E**) Top: Representative images showing calcium signal (maximum z-projection) of individual neurons from imaging video from day 1 (left) and day 2 (right) of recording. Red dots: 8 identified and aligned Vglut2^LHA-LHb^ neurons. Middle: Raster plot of the average individual activity of the 8 Vglut2^LHA-LHb^ neurons on day 1 and day 2 of recording. Bottom: The average calcium signal of the 8 Vglut2^LHA-LHb^ neurons on day 1 and day 2 of recording.

(**F**) Average calcium trace (red trace) of CS-modulated Vglut2^LHA-LHb^ neurons (purple cluster, Fig. 5F-G) and freezing behavior (minimum 1 sec immobility shown in light blue) from one representative mouse. The entire session of the fear conditioning experiment on day 1 (top) and day 2 (bottom) is shown. Dark blue bars: CS (tone), red bars: US (foot shock).

(**G**) The average calcium signal on day 2 during freezing periods (mouse immobility for at least 1 sec), which occur outside the tone presentation (intertrial interval, ITI), is significantly lower compared to the average calcium signal during freezing in the CS period (CS+) for CS-modulated LHA Vglut2 neurons (Vglut2^LHA^) and for CS-modulated LHA-LHb Vglut2 neurons (Vglut2^LHA-LHb^).

(**H**) Normalized response (response scaled to the peak response over the entire calcium trace) to reward vs shock of individual neurons from all imaged mice (each mouse and corresponding neurons assigned a unique color-code). Left: Vglut2+ neurons in the LHA (data from 4 mice). Right: Vglut2^LHA-LHb^ neurons (data from 3 mice).

Scale bars: 1 mm in (D right), 500 µm in (D left), 20 µm in (E right) and 100 µm in (E left). * *P* < 0.001. Line shading: mean ± 95% confidence interval.

**Supplementary Videos**

**Supplementary Video 1. Optogenetic activation of Vglut2/Vgat^GPi-LHb^ neurons in the real-time place preference assay.**

Video (4x speed) showing behavior of the mouse during activation Vglut2/Vgat^GPi-LHb^ neurons (GPi-LHb stimulation) on the right-side compartment. Data presented in Fig. 3A-C, G.

**Supplementary Video 2. Optogenetic activation of Vglut2^LHA-LHb^ neurons in the real-time place preference assay.**

Video (4x speed) showing behavior of the mouse during activation Vglut2^LHA-LHb^ neurons (LHA-LHb stimulation) on the right-side compartment. Data presented in Fig. 3D-G.

**Supplementary Video 3. Calcium imaging of GCaMP6s-expressing LHA Vglut2 neurons in the fear conditioning assay.**

Video relating to data presented in Fig. 4.

Video (2x speed) shows calcium signal on Day 1 and Day 2 in in the fear conditioning assay. Arrowheads point to three selected neurons, showing the extracted calcium trace below. Dashed lines indicate the start of the tone (CS), and the start of the foot shock (US), respectively.

**Supplementary Video 4. Calcium imaging of GCaMP6m-expressing Vglut2^LHA-LHb^ neurons in the fear conditioning assay.**

Video relating to data presented in Fig. 5.

Video (2x speed) shows calcium signal on Day 1 and Day 2 in in the fear conditioning assay. Arrowheads point to three selected neurons, showing the extracted calcium trace below. Dashed lines indicate the start of the tone (CS), and the start of the foot shock (US), respectively.
