# Supplementary material for: A hypothalamus-habenula circuit controls aversion
Source: Mol Psychiatry. 2019 Feb 12;24(9):1351–68. doi: 10.1038/s41380-019-0369-5 (PMC6756229; doi:10.1038/s41380-019-0369-5)
Supplement: Supplementary file 2 — supplementary table 1 [file 41380_2019_369_MOESM2_ESM.pdf]

Supplementary Table 1

Paradigm	Mouse genotype	Number of mice	Virus	Volume (nl)	Inj. site	Optical fiber placement	Stimulation parameters	Effect	c-fos	Figure
Place preference	Wild-type	6	AAV DIO EYFP	200	LHA	LHb (terminals)	60 Hz, 1ms pulse, 10mw, 447nm laser	—		Fig.3G
	Vglut2-Cre	16*	AAV DIO ChR2-mCherry	70	LHA	LHb (terminals)	60 Hz in 10 mice, 30 Hz in 6 mice 1ms pulse, 10mw, 447nm laser	Negative valence		Fig.3D-F, G
	Vglut2-Cre/Vgat-Flpo	6*	AAV Cre-on/Flpo-on ChR2-EYFP	200	GPi	GPi (cell bodies)	30 Hz, 5ms pulse, 10mw, 447nm laser	—	Fig. S3A-F	Fig.3A-C, G
	Vglut2-Cre	6	AAV DIO ChR2-mCherry	70	GPi	LHb (terminals)	60 Hz, 1ms pulse, 10mw, 447nm laser	—		Fig.3G
	Wild-type	7	AAV CAG ChR2-mChery	70	GPi	LHb (terminals)	60 Hz, 1ms pulse, 10mw, 447nm laser	—		Fig.3G
	Sst-Cre	22	AAV DIO ChR2-mCherry	200	GPi	LHb (terminals)	60 Hz, 1ms pulse, 10mw, 447nm laser	—		Fig.3G
	Sst-Cre	6	Retrograde AAV DIO ChR2-mCherry	200	GPi	GPi (cell bodies)	30 Hz, 5ms pulse, 10mw, 447nm laser	—	Fig. S3G-L	Fig.3G
Probabilistic switching	Vglut2-Cre	5*	AAV DIO ChR2-mCherry	70	LHA	LHb (terminals)	30 Hz, 5ms pulse, 10mw, 447nm laser	Negative valence		Fig.3J, L, N
	Vglut2-Cre/Vgat-Flpo	5*	AAV Cre-on/Flpo-on ChR2-EYFP	200	GPi	GPi (cell bodies)	30 Hz, 5ms pulse, 10mw, 447nm laser	—	Fig. S3A-F*	Fig.3K, M, O

* 5 mice used in both paradigms
