# Supplementary figures and images for: A hypothalamus-habenula circuit controls aversion
Source: Mol Psychiatry. 2019 Feb 12;24(9):1351–68. doi: 10.1038/s41380-019-0369-5 (PMC6756229; doi:10.1038/s41380-019-0369-5)

A

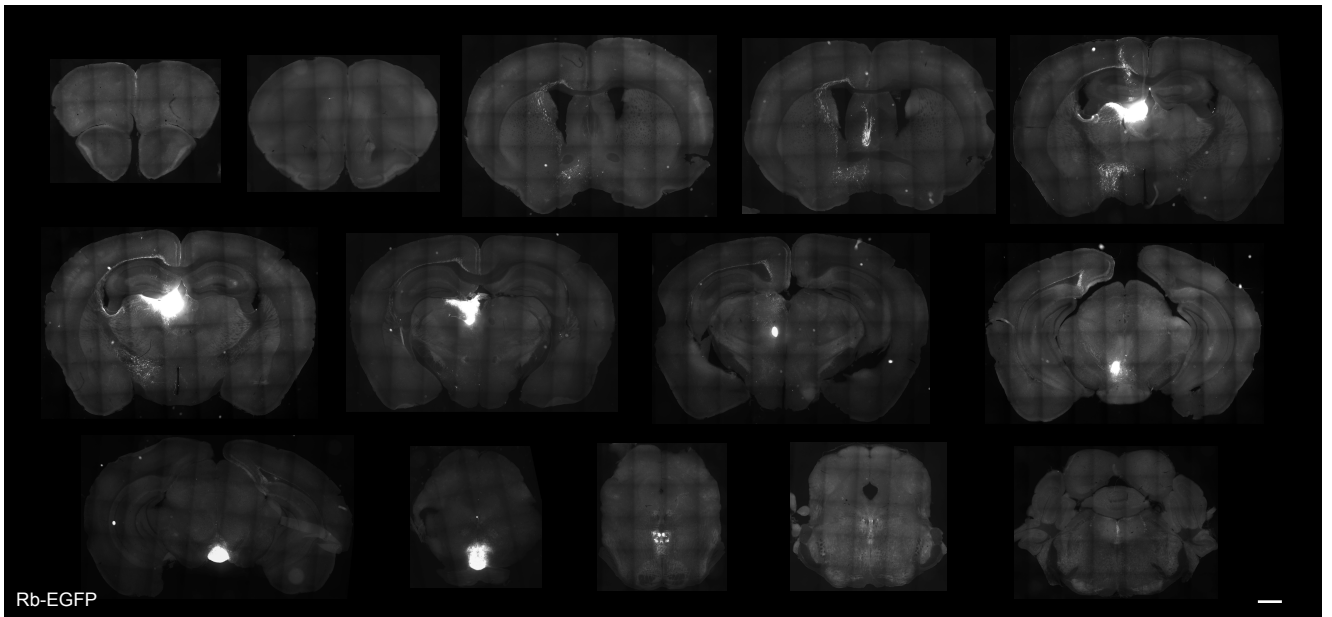

B

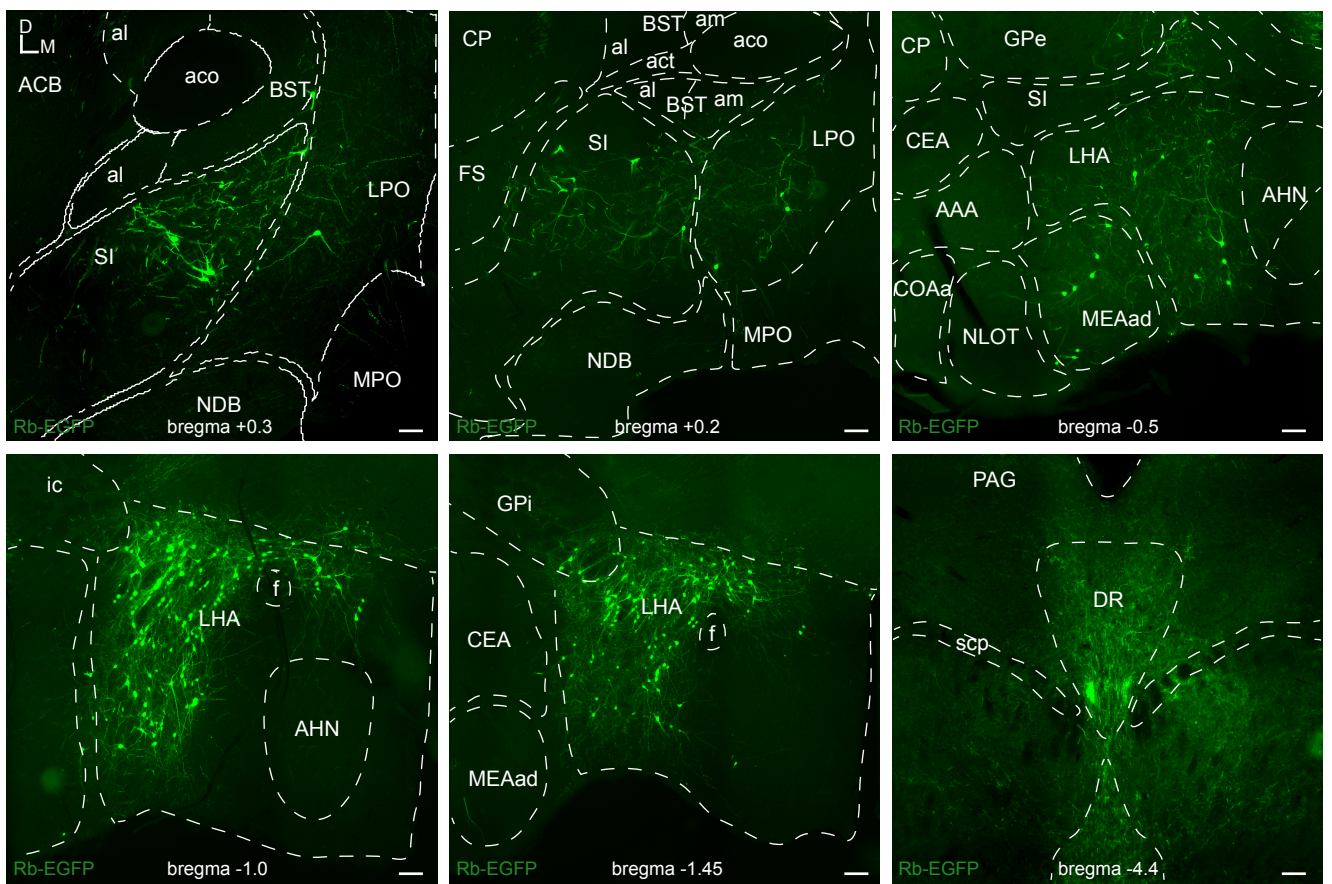

Supplement: Supplementary file 7 — supplementary figure 1 [file 41380_2019_369_MOESM7_ESM.pdf]

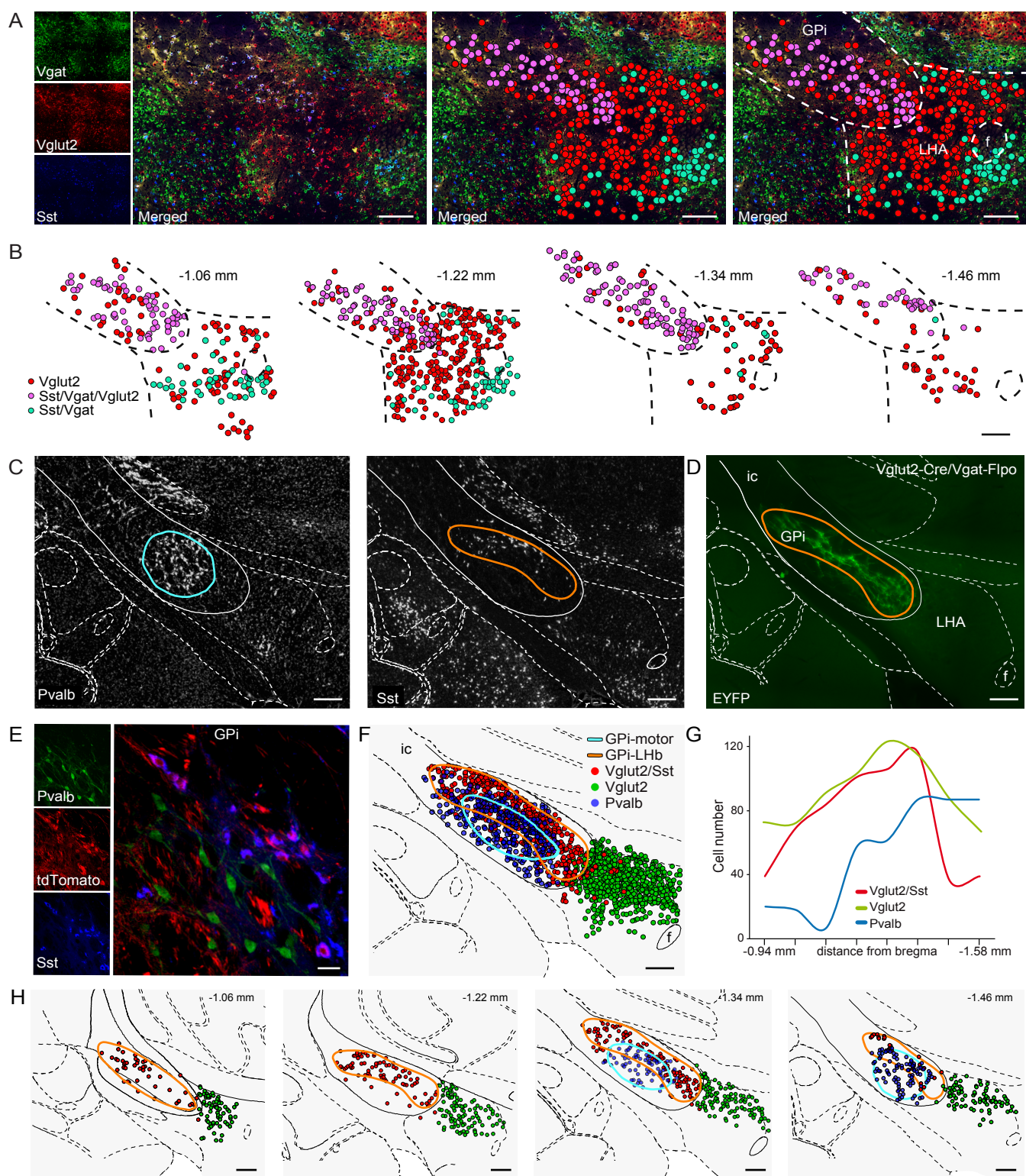

Supplement: Supplementary file 8 — supplementary figure 2 [file 41380_2019_369_MOESM8_ESM.pdf]

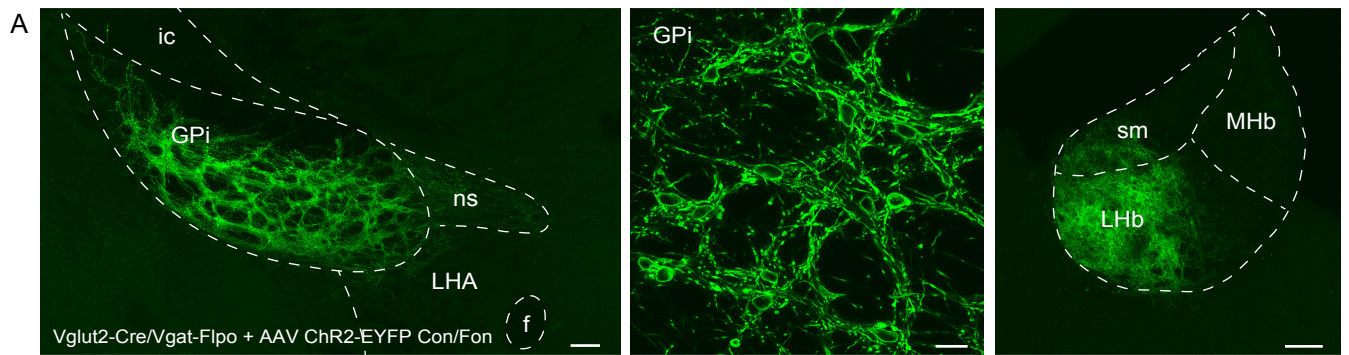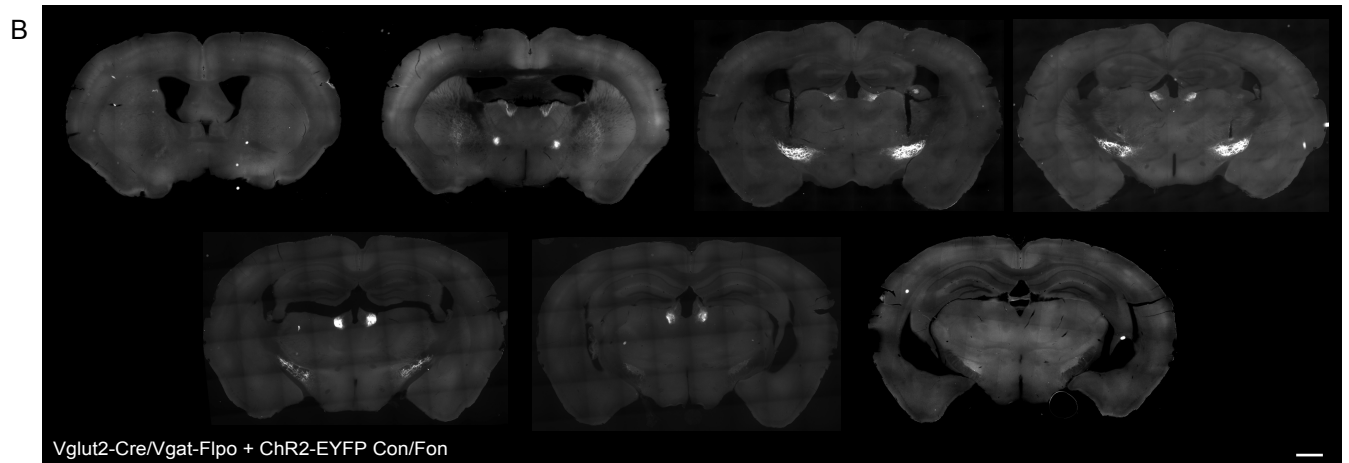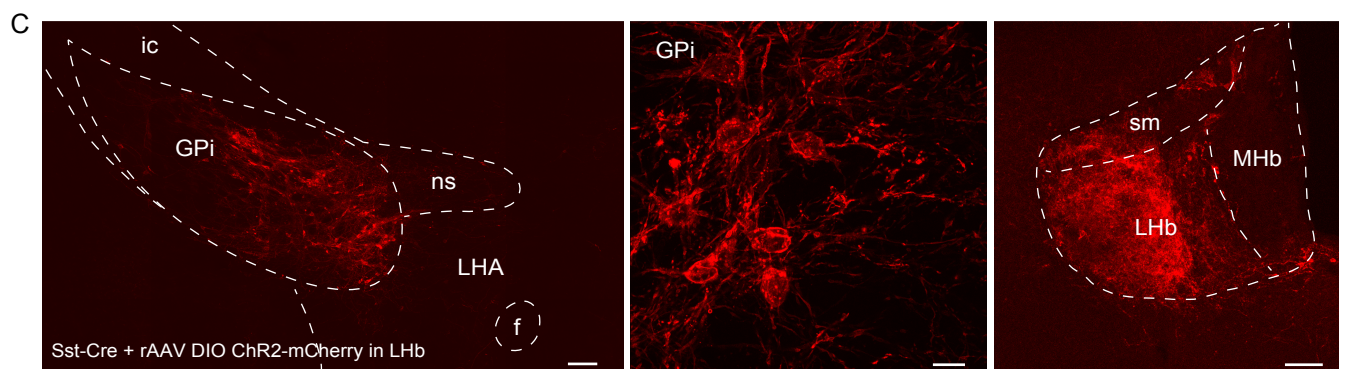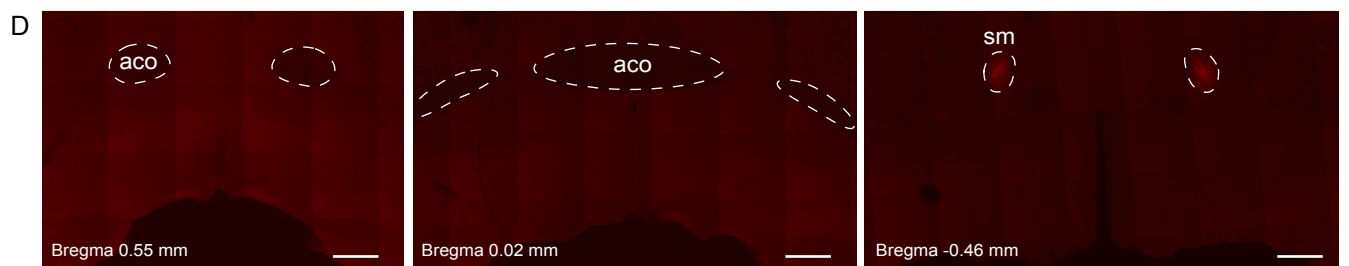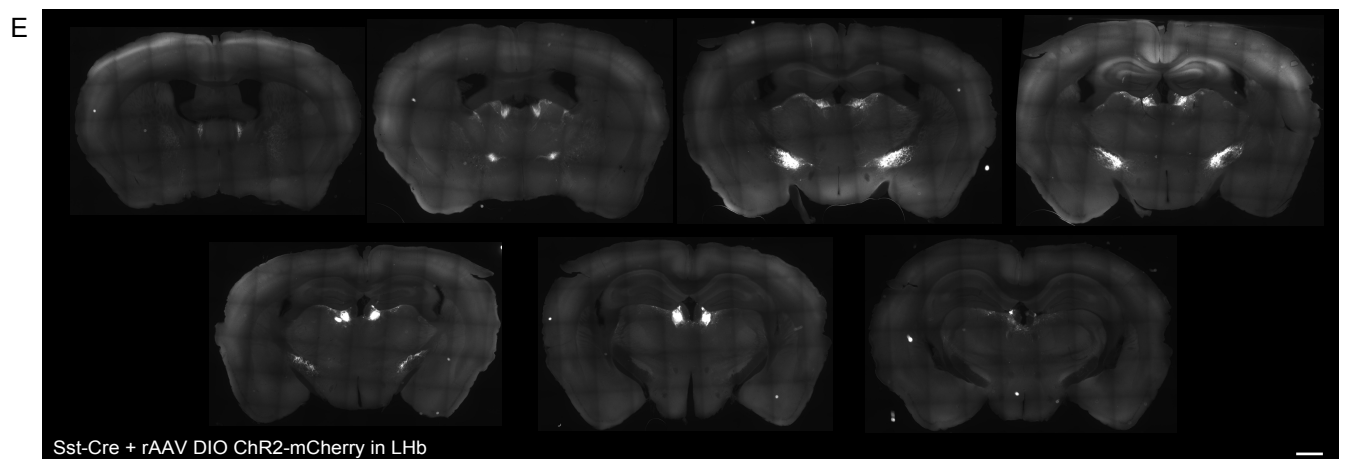

Supplement: Supplementary file 9 — supplementary figure 3 [file 41380_2019_369_MOESM9_ESM.pdf]

A

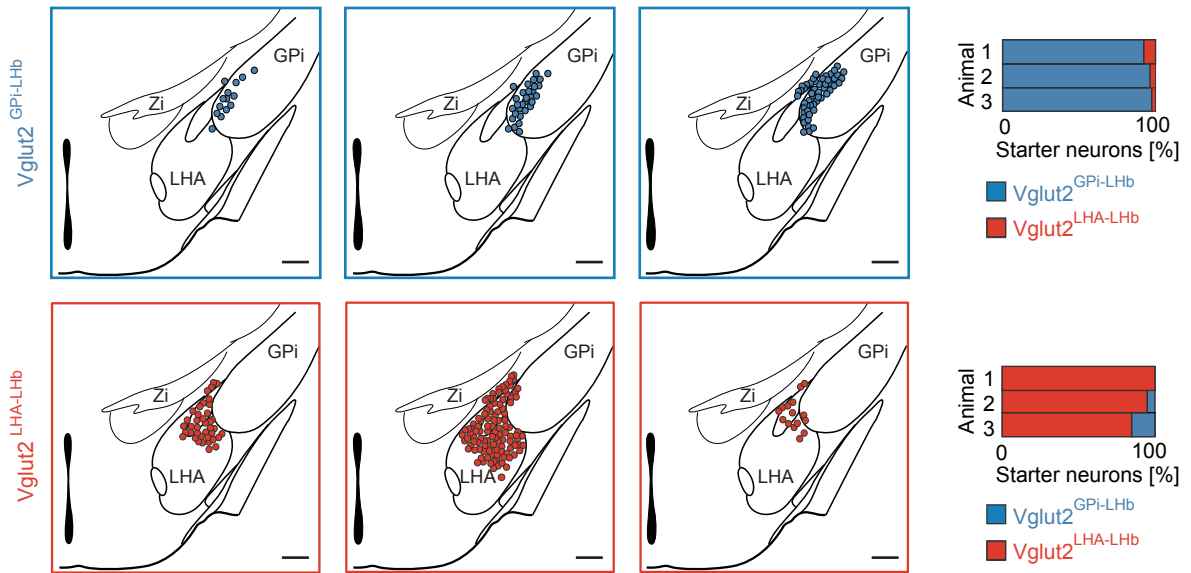

B

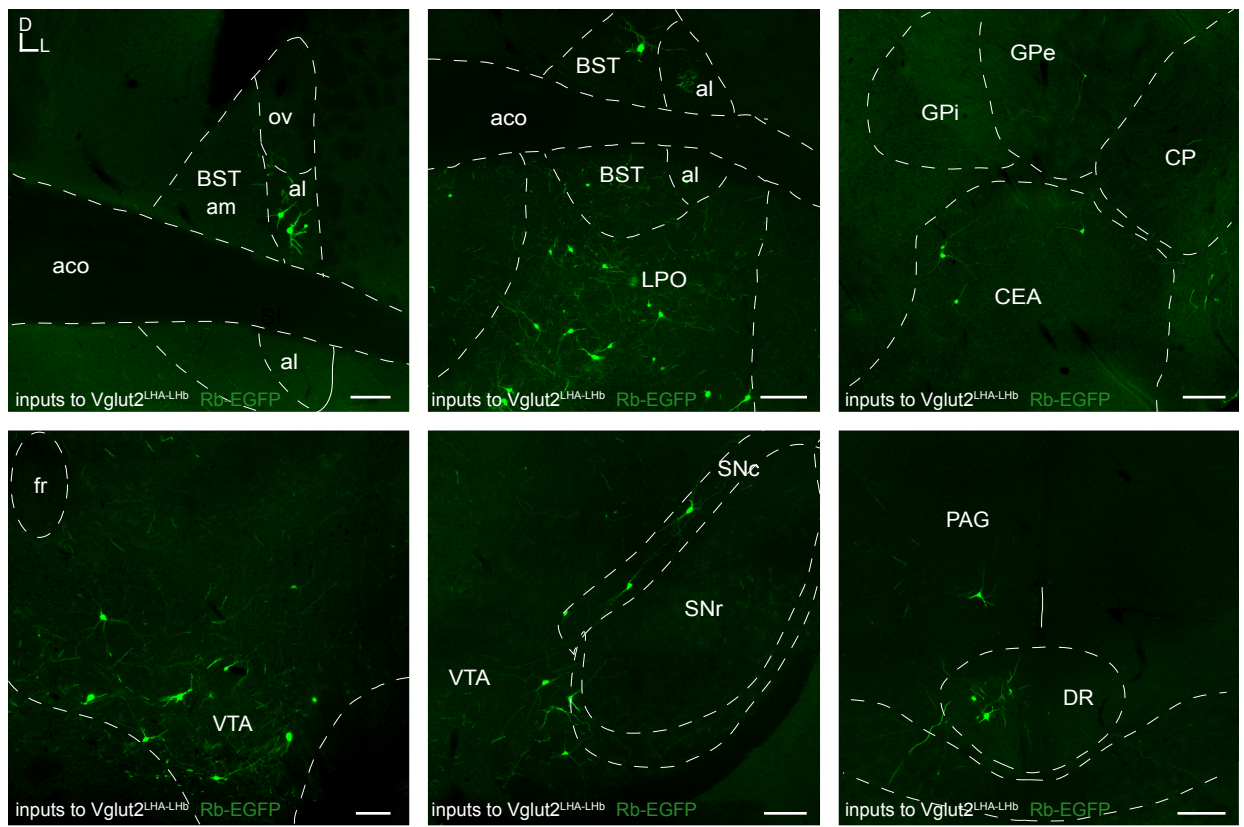

C

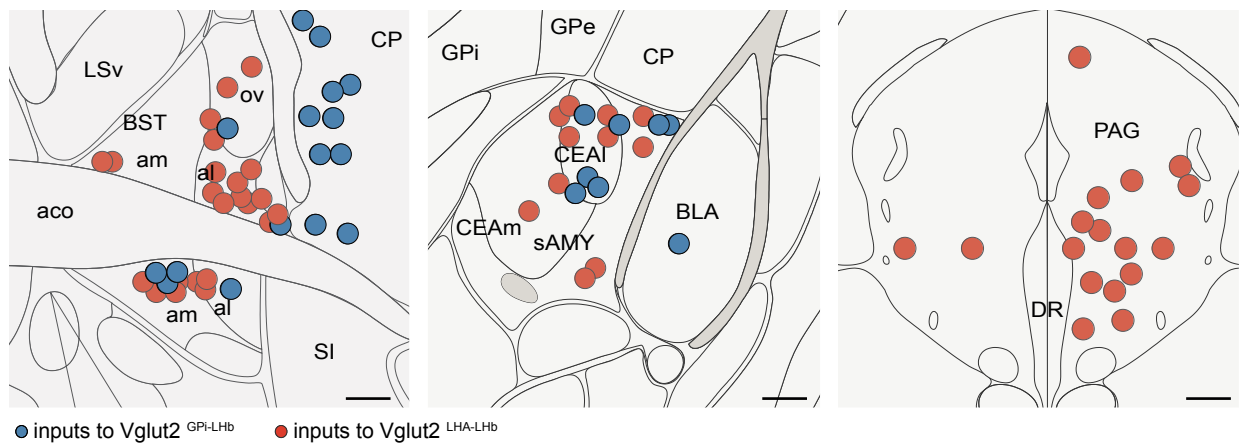

Supplement: Supplementary file 10 — supplementary figure 4 [file 41380_2019_369_MOESM10_ESM.pdf]

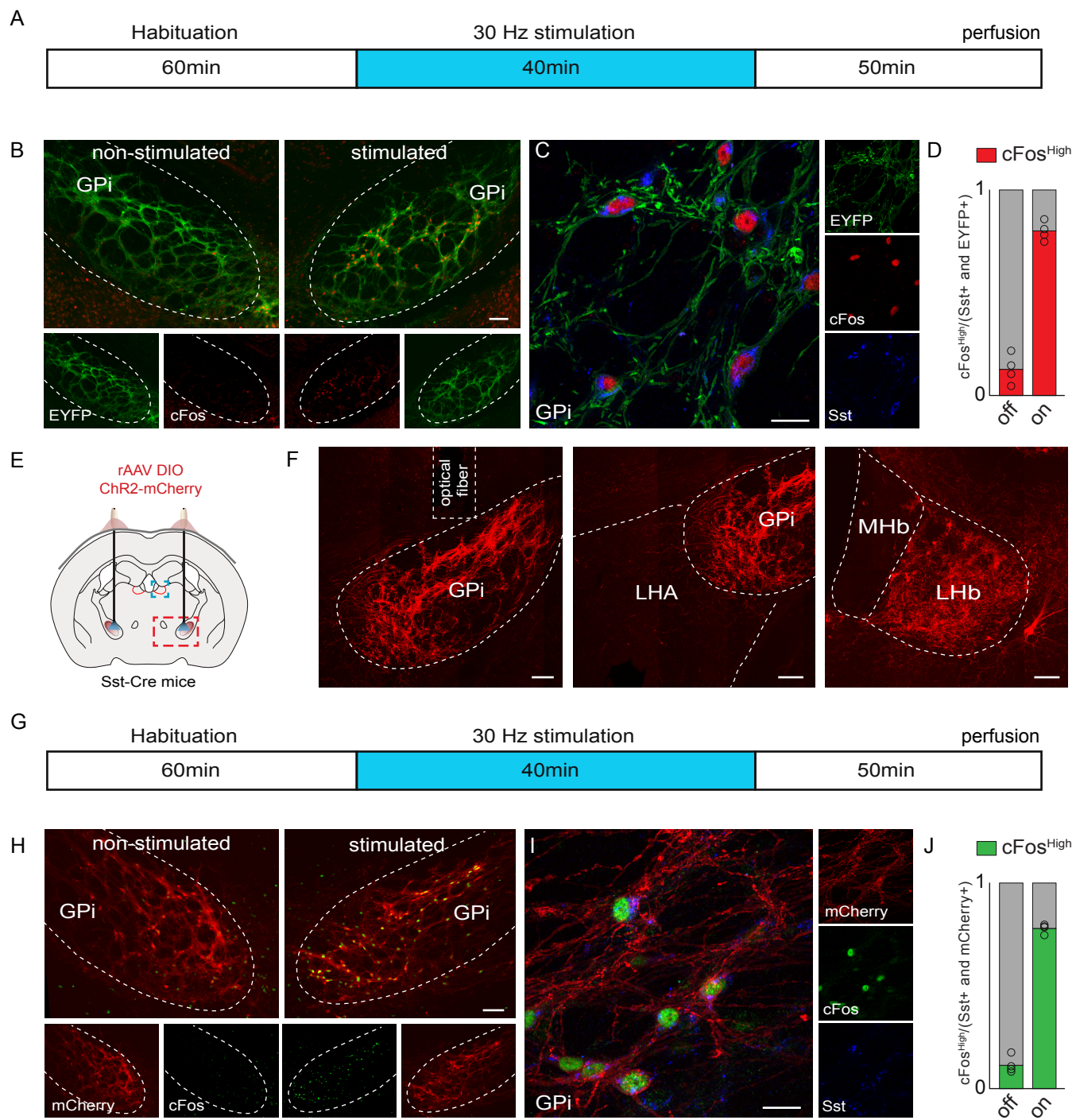

Supplement: Supplementary file 11 — supplementary figure 5 [file 41380_2019_369_MOESM11_ESM.pdf]

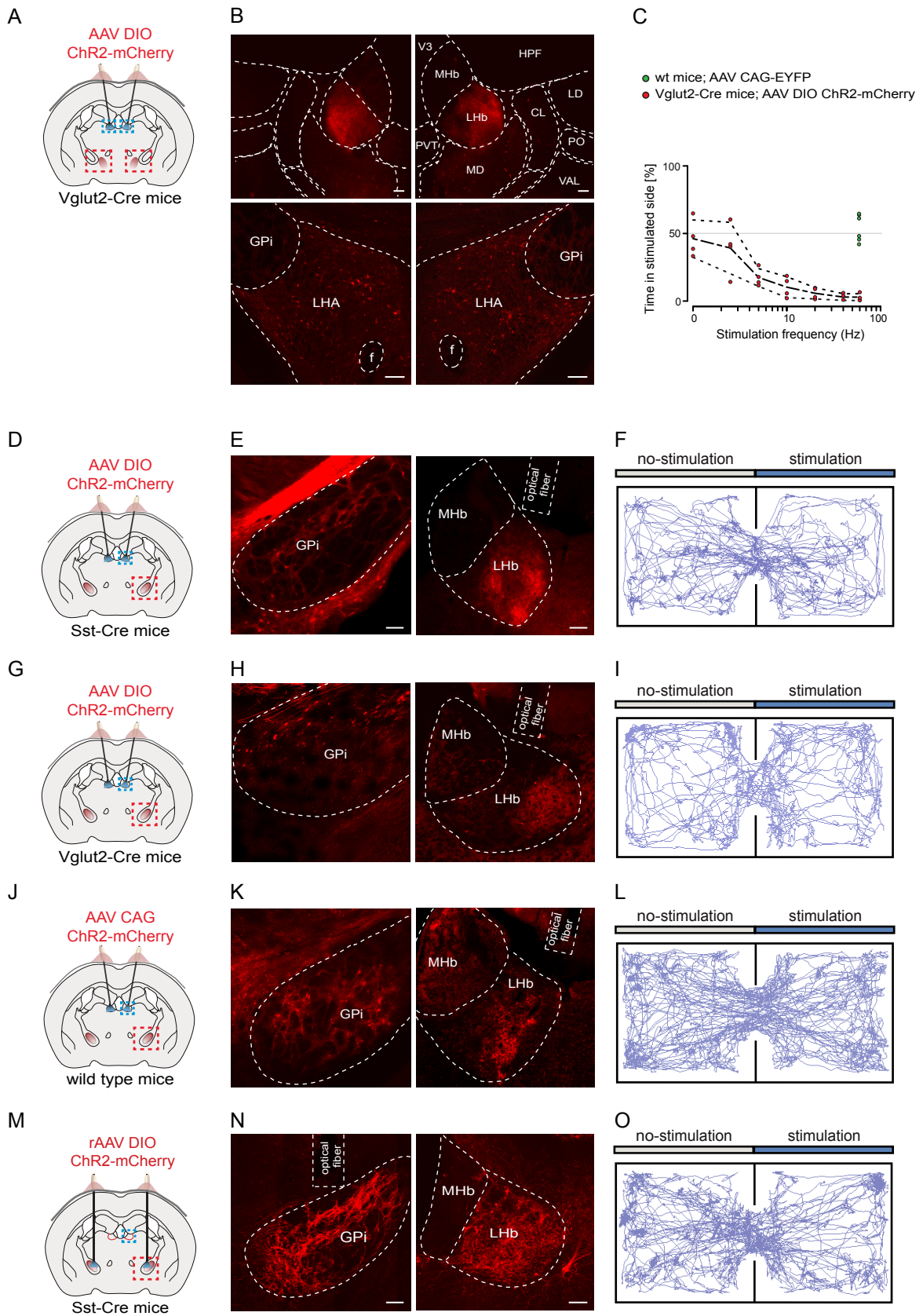

Supplement: Supplementary file 12 — supplementary figure 6 [file 41380_2019_369_MOESM12_ESM.pdf]

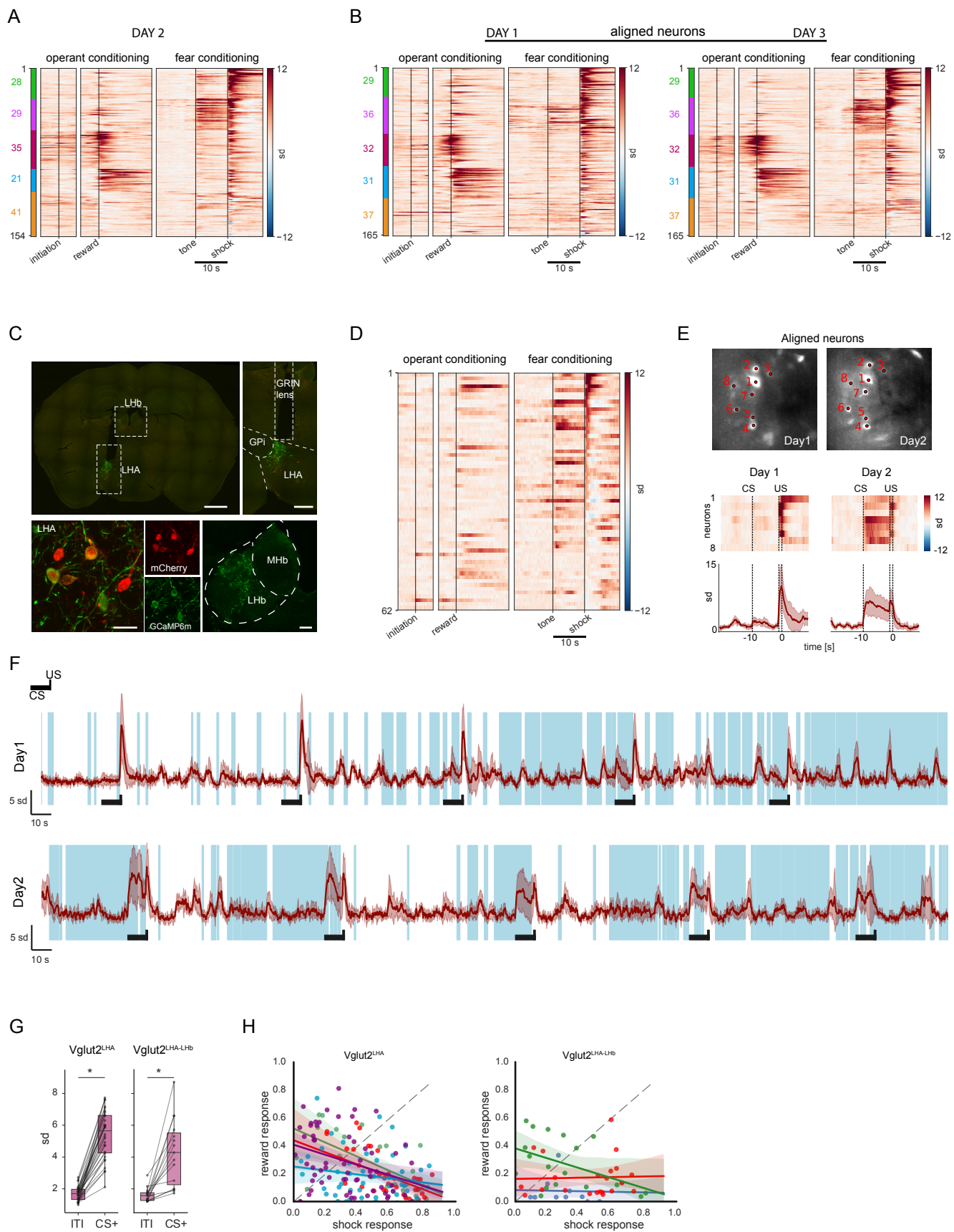

Supplement: Supplementary file 14 — supplementary figure 8 [file 41380_2019_369_MOESM14_ESM.pdf]
