# Supplementary material for: A hypothalamus-habenula circuit controls aversion
Source: Mol Psychiatry. 2019 Feb 12;24(9):1351–68. doi: 10.1038/s41380-019-0369-5 (PMC6756229; doi:10.1038/s41380-019-0369-5)

A

Training and testing schedule in operant and fear conditioning

training 1-2 weeks	test day 1:		test day 2:		test day 3:	
operant conditioning 45min	operant conditioning 45min	fear conditioning 5 CS/US	operant conditioning 45min	fear conditioning 5 CS/US	operant conditioning 45min	fear conditioning 5 CS/US

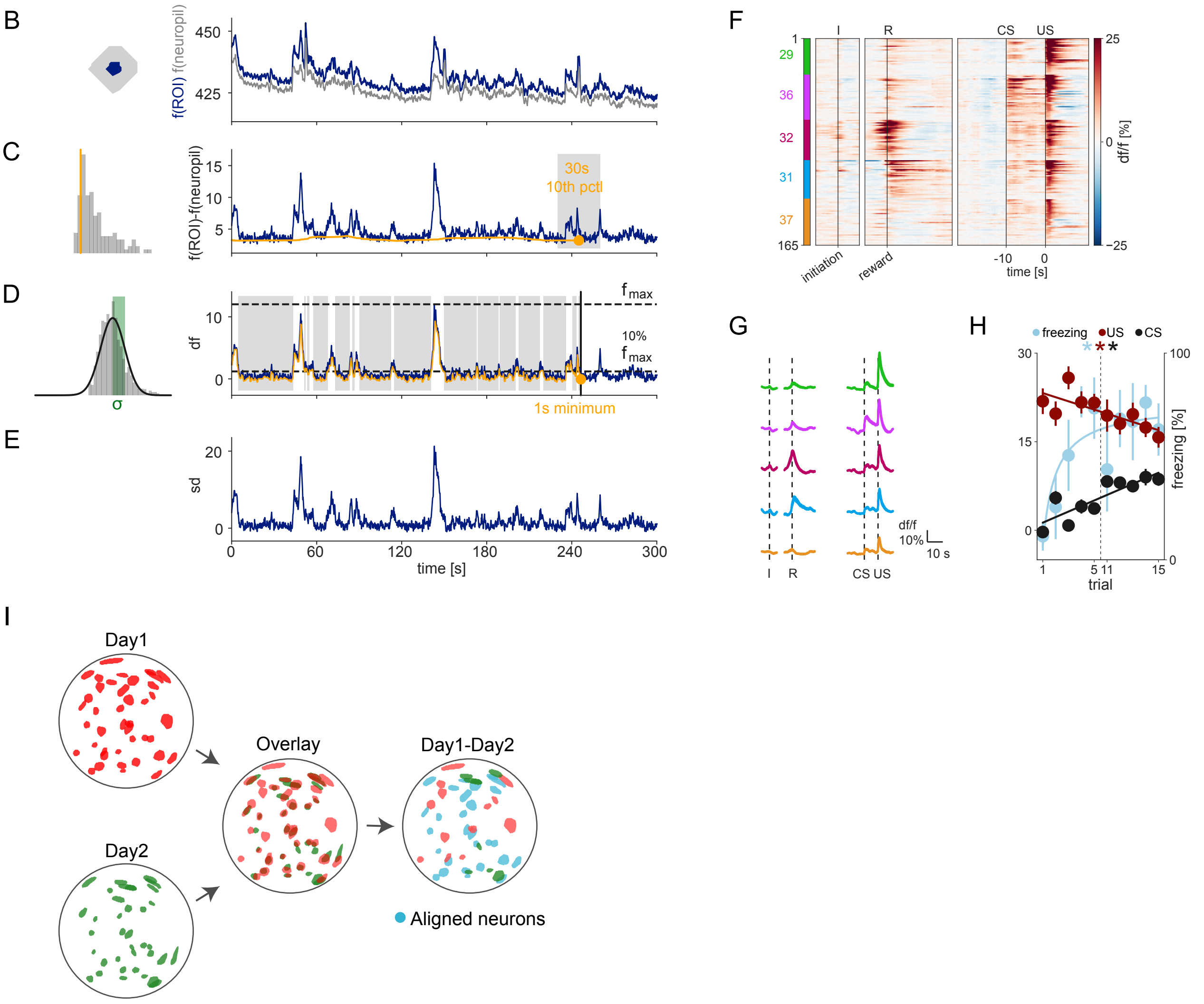

Supplement: Supplementary file 13 — supplementary figure 7 [file 41380_2019_369_MOESM13_ESM.pdf]
